# Non-Traditional Luminescent Polyurethanes of *n*–π Electron Hybrid Structures with Varying Separation of Aromatic Rings

**DOI:** 10.1021/acsapm.5c02091

**Published:** 2025-09-12

**Authors:** Ziwei Wang, Yingqi Li, Han Zhang, Nan Jiang, Jiawei Xu, Dongxia Zhu, Martin R. Bryce

**Affiliations:** † Key Laboratory of Preparation and Applications of Environmental Friendly Materials, 47825 Jilin Normal University, Changchun 130103, China; ‡ Ministry-of-Education Key Laboratory of Numerical Simulation of Large-Scale Complex System (NSLSCS) and School of Chemistry and Materials Science, Nanjing Normal University, Nanjing 210023, China; § Key Laboratory of Nanobiosensing and Nanobioanalysis at Universities of Jilin Province, Department of Chemistry, 47821 Northeast Normal University, 5268 Renmin Street, Changchun, Jilin Province 130024, P. R. China; ∥ Department of Chemistry, 3057 Durham University, Durham DH1 3LE, U.K.

**Keywords:** nontraditional luminescence, polyurethanes, multicolor emission, aggregation, information transmission

## Abstract

Detailed structure–activity relationships and molecular design criteria for nonconventional and nonconjugated luminescent polymers are still ambiguous. This work reports *n*–π electron hybrid polyurethane derivatives (**PUs**) with varying separation of benzene rings in the backbone. Experimental and computational results show that although through-bond conjugation is not the main source of luminescence, the separated benzene rings still modulate the photophysical properties by affecting the molecular conformation and electron transport. Meanwhile, hydrogen bonds and *p*–π* interactions exert a significant influence on the emission wavelengths by regulating the aggregated-state structures. In **PU1**, the excited state is predominantly charge transfer. With an increase in the number of benzene rings along the chain, the energy gap between the acceptor π and π* orbitals is reduced; alongside these effects, the modulation from hydrogen bonds and *p*–π* interactions promotes a transition to LE-dominated states in **PU3**, which incorporates terphenyl units. Proof-of-concept applications are demonstrated in information transmission and colorful displays. This work enhances the understanding of the mechanisms underlying the excitation-dependent properties of nonconventional polymers and provides a strategy for tuning and exploiting their intrinsic luminescence.

## Graphical abstract

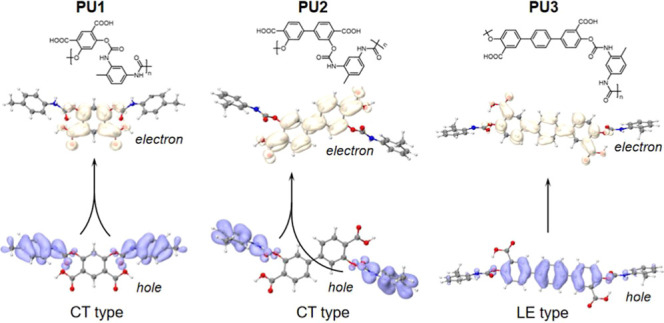

## 1 Introduction

In the long history of mankind, the exploitation of light has played a crucial role in humans’ perception of the outside world and the advancement of society. Organic luminescent materials (OLMs) have attracted much attention in many fields such as optoelectronic devices, response sensing, and biomedicine due to their unique and tunable optical and structural properties.^1–5^ The molecular design of traditional luminescent materials mostly relies on through-bond conjugation (TBC), wherein an expanded π-conjugated structure generated by covalent bonds produces electronic transitions which result in emission.^6–9^ However, traditional OLMs usually have high manufacturing costs which may require the use of expensive transition metal catalysts, and they often suffer from molecular aggregation due to π–π stacking which results in luminescence quenching by facilitating nonradiative energy dissipation, and the materials can be difficult to process.^10–12^ In recent years, nonconventional luminescent polymers (NCLPs)—a class of nonconjugated or weakly conjugated polymers that exhibit intrinsic luminescence without relying on extended π-conjugated backbones through covalent bonds—have emerged as a promising alternative to OLMs. Unlike traditional luminescent polymers dominated by TBC, NCLPs derive their luminescence mainly from through-space conjugation (TSC) of electrons (e.g., spatial overlap of n or π orbitals between separated chromophores) and noncovalent interactions (e.g., hydrogen bonds, π–π interactions, and *p*–π* interactions),^13–17^ which fundamentally expands the design paradigm of luminescent materials beyond the constraints of covalent conjugation.^18–20^

NCLPs can be categorized into three main types based on their electronic structures: (i) pure *n*-electron structures, like polysiloxanes, polyethers, and polythioethers;^21,22^ (ii) pure π-electron structures with separated aromatic rings, for example, diphenylmethane, polyphenylallenes, and diphenylethane;^23,24^ and (iii) (*n*–π)-electron hybrid structures such as cyclohexadienones, polyketones, and maleimides.^25–28^ Notably, the fundamental significance of NCLPs lies in (i) challenging the traditional π-conjugation paradigm by revealing roles of TSC and noncovalent interactions; (ii) overcoming aggregation caused quenching (ACQ), high cost, and poor processability of conventional materials; and (iii) enabling tunable luminescence for optoelectronics, bioimaging, and information encryption. Compared with small molecules, polymers can have advantages such as multivariable structure regulation, simple synthesis at low cost, customizable photophysical and mechanical properties, easy large-scale synthesis, and solution processing.^16,29–31^ Thus, research on NCLPs is of practical significance to guide the theory establishment and application development of OLMs. However, due to concerns about the influence of trace impurities and the cost of computational resources, investigations of the luminescence mechanism have mainly focused on small molecule systems, and there is still a long way to go to establish unified criteria for their remarkable luminescent behavior, especially for nontraditional luminescent polymers. More examples of macromolecules/polymers are urgently needed to decipher the effects of NCLPs’ internal electronic structure and aggregation behavior on the emission mechanisms, thereby helping to establish universal molecular design principles.^32–34^

In the past few years, continuous experimental and theoretical inquiries have demonstrated that the abnormal luminescence of NCLPs is generally not from unknown impurities in the raw material, nor from the byproducts of the polymerization reaction, but from the aggregated state of the material itself.^35–37^ In this context, our work on polyurethane (**PU**) derivatives has led to some conclusions: notably, high temperature is conducive to increased intramolecular and/or intermolecular noncovalent interactions such as hydrogen bonds and the formation of good aggregation structures but is not conducive to spatial *n*–π interactions.^38–40^ Based on the above background, in the present study, *n*–π electron hybrid NCLP structures with varying degrees of separation of aromatic rings were designed and synthesized to enrich the experimental and theoretical case studies and to promote the targeted optimization of such materials.

## 2 Results and Discussion

### 2.1 Synthesis

The **PUs** were synthesized by reacting 2,4-toluenediisocyanate and diol monomers 4,6-dihydroxybenzene-1,3-dicarboxylic acid for **PU1** or 3,3-dihydroxy-[1,1-biphenyl]-4,4-dicarboxylic acid for **PU2** or 4,4″-dihydroxy-[1,1′:4′,1″-terphenyl]-3,3″-dicarboxylic acid for **PU3** in one-pot reactions (Figure 1). The detailed synthetic routes and structural characterization are shown in the Supporting Information. The ^1^H NMR spectra (Figure S1) are consistent with their structures. The Fourier transform infrared (FT-IR) spectra (Figure S2) and gel permeation chromatography data further validated the successful synthesis of the **PUs**. *M*_w_ values of the products were within the range of 7.1 × 10^3^–2.5 × 10^5^ g mol^–1^ (Figure S3 and Table S2). It was of immediate interest that under 365 nm UV irradiation, **PU3** showed blue fluorescence, **PU2** yellow, and **PU1** orange. Their QYs follow the sequence **PU3** (2.1%) > **PU1** (1.7%) > **PU2** (1.1%), with lifetimes of 3.11, 2.42, and 4.62 ns, respectively, aligning with the emission traits (Figures 2 and S4a and Table S1).

**Figure 1:**
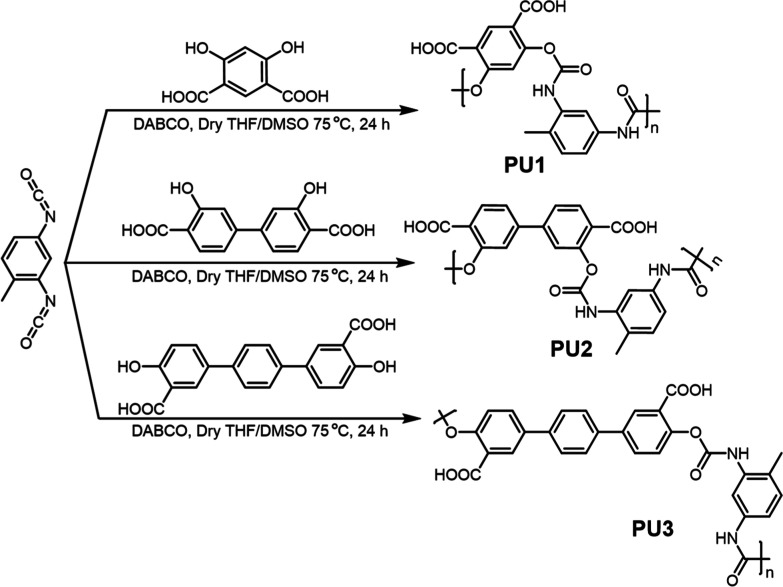
Synthetic routes and chemical structures of **PU** derivatives **PU1**, **PU2**, and **PU3** studied in this work.

**Figure 2:**
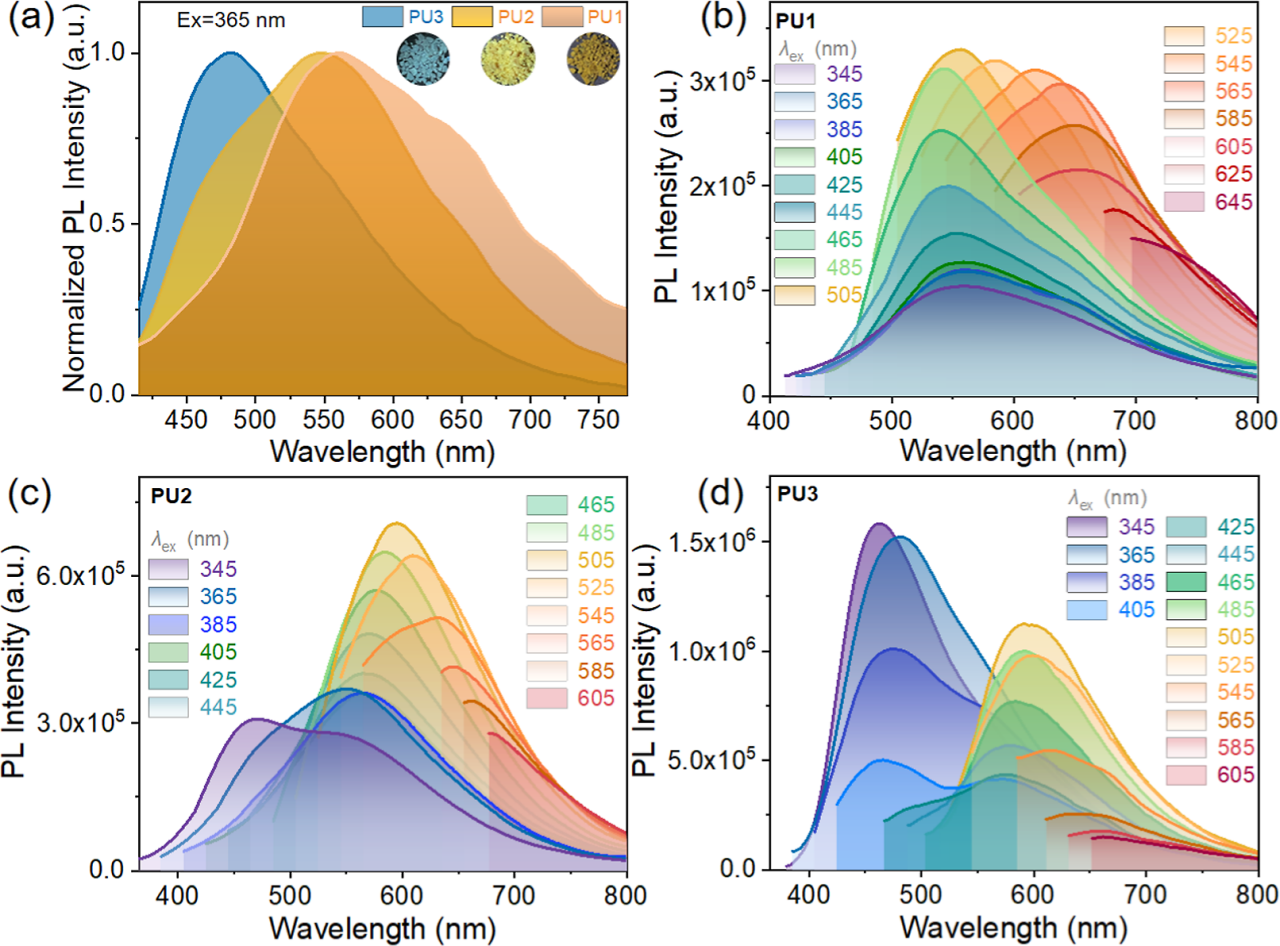
(a) Normalized emission spectra of **PU1**, **PU2**, and **PU3** powder (λ_ex_ = 365 nm); inset: the corresponding fluorescence photographs under 365 nm UV illumination. Photoluminescence (PL) spectra of (b) **PU1**, (c) **PU2**, and (d) **PU3** powders with different excitation wavelengths at room temperature.

### 2.2 Physical Properties

Ultraviolet–visible (UV–vis) absorption spectra show that in the solution (dispersed) state, the **PUs** mainly exhibit a narrow absorption peak at around 275 nm, which comes from benzene ring units (Figure S4b). However, weak acromions can also be observed at 300 nm, especially for **PU1**. It is suspected that aggregates induced by intermolecular interactions also become absorbing species in solution, which is supported by the wide absorption of the **PUs**’ solid (aggregated) samples in Figure S4b. In the aggregated state, **PUs** exhibit a broader range of useable light absorption, which coincides with the emission wavelength (Figure 2a). In addition, as shown in Figure S4c,e, the excitation spectra of **PU1**, **PU2**, and **PU3** monitored at different emission peaks exhibit distinct profiles. The excitation spectrum of **PU1** shows relatively clear excitation peaks at specific wavelengths, indicating that its response to excitation light is relatively more “focused” on specific wavelength bands. **PU2** shows additional excitation peaks, implying that it can respond to more diverse excitations and possibly has more energy absorption channels. The excitation peaks of **PU3** are more dispersed, reflecting a broader-spectrum excitation response with absorption across different wavelength bands of excitation light. These results demonstrate that differences in the molecular structure or aggregation state among the three samples lead to diverse excitation responses.

To better understand the aggregation process, **PUs**/DMSO solutions (0.1–3 mg mL^–1^) with gradient concentrations were prepared. Figure S5 shows that progressive aggregation leads to absorption enhancement and red-shifted characteristics by increasing the concentration of the **PUs**. However, curiously, as shown in Figure S5c, at high concentrations, **PU3** has a stronger shoulder absorption and a longer wavelength tail absorption, which are different from the solid sample absorption properties (Figure S4b). To probe these features, we studied their emission spectra. As shown in Figure S6, the emission peak of the **PUs** varies greatly in the range of 0.1–3 mg mL^–1^. For **PU2**, the emission hardly changes as the concentration increases. For **PU1**, initially, (0.1–2.5 mg mL^–1^) its emission peak changes very little, but at 3 mg mL^–1^, the peak has a large red shift (32 nm). To gain an in-depth understanding of the ground-state electronic structure of **PUs**, cyclic voltammetry (CV) was employed to study the highest occupied and lowest unoccupied molecular orbital (HOMO and LUMO, respectively) levels of the **PUs**. Figure S5d,e displays the CV curves of **PU1**, **PU2**, and **PU3**, along with the correspondingly calculated HOMO and LUMO levels. Their variation trend coincides with the emission spectra of the **PUs** (Figure 2a).

The emission peak of **PU3** gradually red-shifts and widens with increasing concentration (with a maximum red shift of 64 nm), which is likely associated with the evolution of its aggregated structures at the nanoscale. Scanning electron microscopy (SEM) images (Figure S7) reveal that, compared with the loosely dispersed low-molecular-weight diol monomers, the polymer products exhibit distinct aggregated morphologies at the micron scale. Meanwhile, concentration-dependent monitoring (Figures S8–S13) visually demonstrates the microscale morphological evolution of **PU3** from dispersed to aggregated states, which may indirectly reflect the changes in the nanoscale aggregation behavior. These results collectively imply that the red-shifted emission of **PU3** with increasing concentration is probably related to the formation of smaller aggregates (beyond the resolution of SEM) and the more complex spatial electron conjugation processes in such nanoscale aggregated states, which are intrinsic to the polymer’s aggregation behavior.

To verify this conjecture, excitation light of different energies was used to collect the emission spectra of the aggregated powder samples of the **PUs**, as shown in Figure 2. With the change of excitation energy, **PU1** always presents a single-peak emission (Figure 2b). For **PU2**, high energy (short wavelength) excitation shows bimodal emission characteristics, while low energy (long wavelength) excitation shows unimodal emission (Figure 2c). For **PU3**, high energy excitation presents unimodal emission, while when excited with low energy, the acromion of **PU3** gradually increases, leading to bimodal emission characteristics (Figure 2d). Clearly, the **PUs** exhibit varying degrees of excitation-dependent emission characteristics. Against the backdrop of traditional π-conjugated materials suffering from ACQ, the fluorescence microscopy images in Figure 3 validate the NCLPs’ multicolor emission under different excitation wavelengths. Specifically, **PU1** shows transitions from orange-brown to green and then to yellowish; **PU2** switches from weak yellow to bright green and subsequently to pale yellow; and **PU3** exhibits evolution from cyan-blue to intense green and then to orange. These wavelength-dependent color tunings directly reflect the NCLPs’ ability to modulate luminescence via excitation energy matching. However, detailed investigations into such excitation-dependent characteristics remain scarce. To further explore the distinct photophysical properties arising from the structural differences of the **PUs**, we resorted to theoretical calculations.

**Figure 3:**
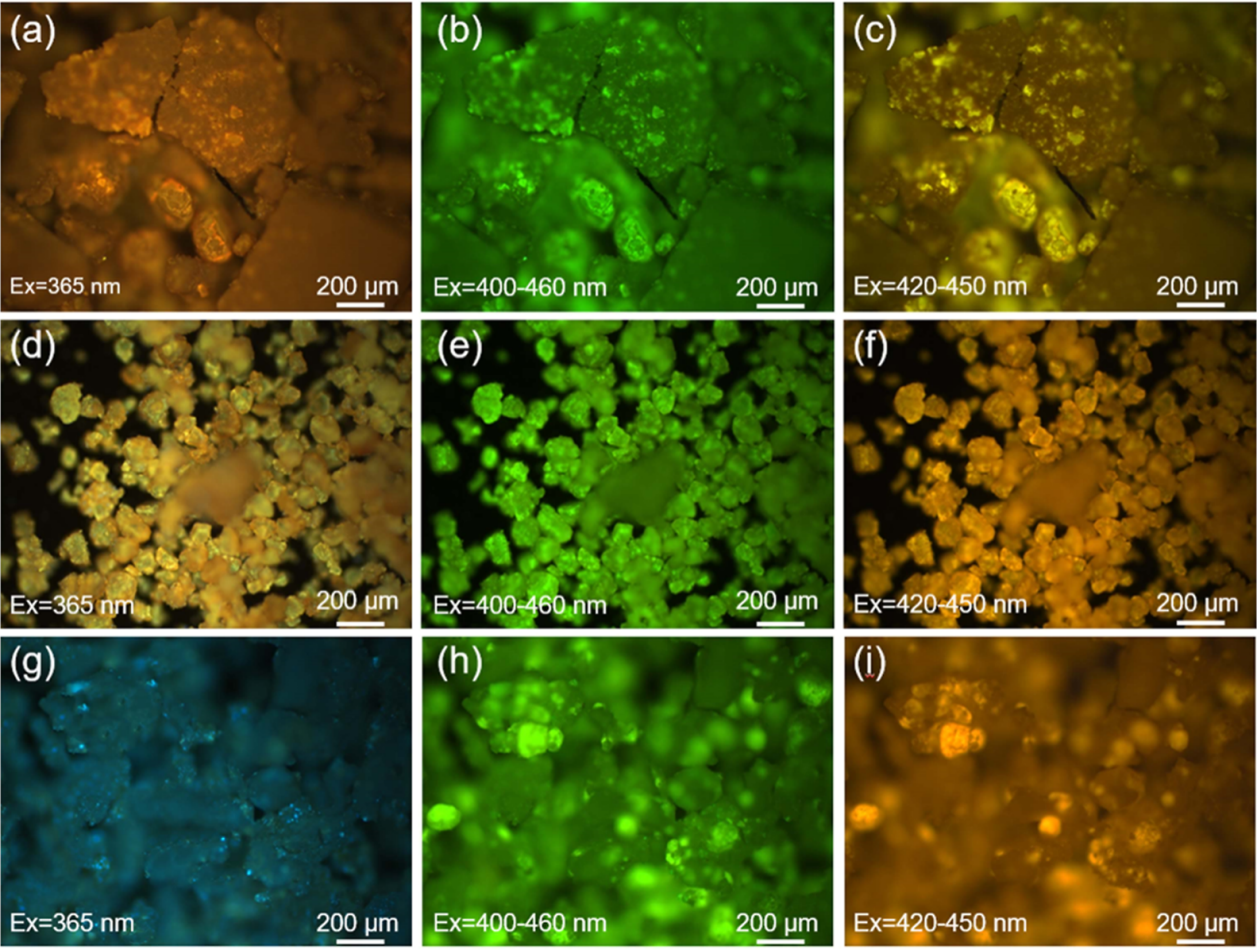
Fluorescence microscopy images of **PU1** (a–c), **PU2** (d–f), and **PU3** (g–i) in the solid state under different excitation wavelengths.

### 2.3 Theoretical Calculations

A computational study based on the photofunctional unit of **PU1** shows that two major conformations are formed by different noncovalent interactions. As shown in Figure 4a,b, both conformations have a charge transfer (CT) feature from a π orbital of D (donor) to a π* orbital of A (acceptor) moieties. Conformation I forms intramolecular hydrogen bonds, bringing a shorter donor···donor distance and local *C*_2_ group symmetry. Meanwhile, conformation II forms through-space *p*–π* interactions and thus has a longer donor···donor distance and local *C*_s_ group symmetry, as well as slightly decreased energy of the S_1_^CT^ state from 4.08 to 3.97 eV. During the formation of *p*–π* interactions, the system promotes the formation of more intermolecular hydrogen bonds by adjusting the bonding mode of hydrogen bonds. A similar phenomenon is also observed in **PU2** (Figure S14). Three-stage molecular dynamics (MD) simulations were performed to investigate the conformation population in the aggregate systems, including (1) MD1: 50.0 ns equilibrium at 298 K; (2) MD2: 50.0 ns equilibrium at synthetic temperature (348 K); and (3) MD3: 50.0 ns annealing to 298 K. The histogram of donor···donor distance during the MD3 simulation (Figure 4c) annealed from 348 K shows that conformation II is the leading form in the **PU1** aggregate system. As shown in Figure 4e and Table S3, under the same synthesis temperature, **PU1** has the largest number of hydrogen bonds, suggesting that **PU1** should have a more compact aggregation state structure, which is largely contributed by stable intermolecular hydrogen bonds in conformation II of **PU1**.

**Figure 4:**
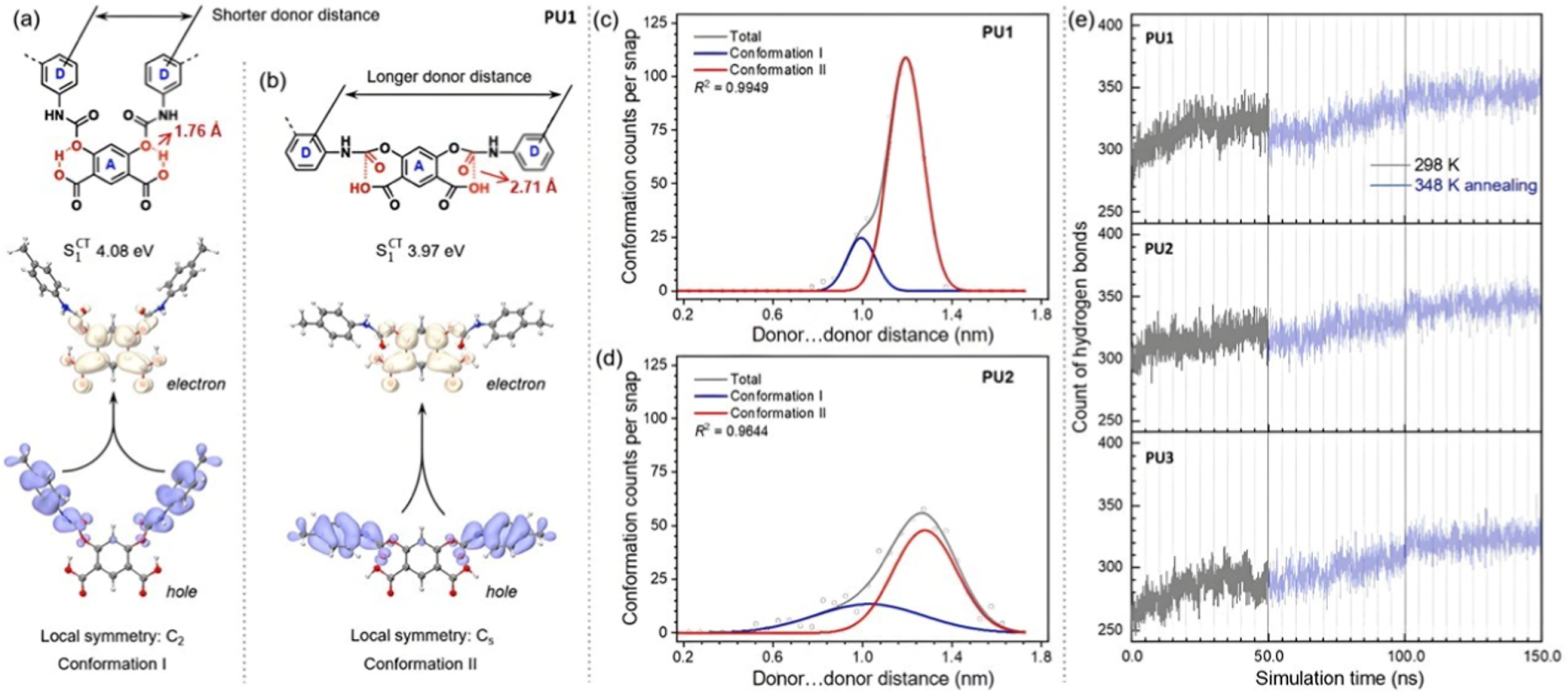
S_1_^CT^ state electron–hole distribution of two major conformations of the **PU1** photofunctional unit with local symmetry of (a) *C*_2_ and (b) *C*_s_ groups. Histograms of the donor···donor distance of (c) **PU1** and (d) **PU2** during the MD3 simulation with a synthetic temperature of 348 K. (e) Counts of hydrogen bond interactions of **PU1**, **PU2**, and **PU3** during 3 × 50.0 ns MD simulations.

However, as shown in Figure 4d, fitting two conformations of **PU2** to the histogram of donor···donor distance has a large deviation compared with **PU1**, suggesting more minor conformations (probably from the rotation of the biphenyl units) may exist in the **PU2** aggregate system, which decreases the ratio of conformation II and results in a slightly reduced photophysical performance than **PU1** (blue-shifted emission and smaller QY) (Table S1). Density functional theory (DFT) calculations based on the photofunctional units of the **PUs** show that as the number of benzene rings increases, the conjugated system within the A part (for **PU1** and **PU2**) and D/A part (for **PU3**) is extended to have a smaller gap between the π/π* orbitals of the acceptor (the local HOMO – LUMO gap of acceptor parts) (Figures 5, S14, and S15), where the energy level of the π orbital is significantly shifted for the higher (marked as red in Figure 5d). As a result, the HOMO in **PU1** or **PU2** is a π orbital of the A moiety but is shifted to a π orbital of the D/A moiety in **PU3**, while the LUMO orbital remains a π* orbital of the D/A moiety (Figure S15). Excitation controlled by a HOMO → LUMO transition shows a CT feature (S_1_^CT^) in **PU1** and **PU2** but a local excitation feature (S_1_^LE^) in **PU3** (Figure 5a). The above results were obtained under a theoretical model, including D–A–D units. To make sure that the model size does not affect the electronic structure, both HOMO – LUMO gap and acceptor gap were further calculated with larger models including (D–A)_2_–D and (D–A)_3_–D units. As shown in Figures S16–S21, energy gaps obtained with (D–A)_*n*_–D (*n* = 1–3) are highly consistent with each other, indicating that the energy level crossing is a general property in the **PUs** regardless of the length of polymer. This is because the twisted conformation formed by a D–A–D unit disables conjugation between two D moieties separated by an A moiety, which is the same for an A–D–A unit, where conjugation between two A moieties separated by a D moiety also does not exist. The LE excitation of an A moiety, or CT excitation on the neighboring D and A moieties, is therefore independent of other units on the same polymer chain and is influenced only by noncovalent interactions, as revealed by MD simulations (Figure 4c,d).

**Figure 5:**
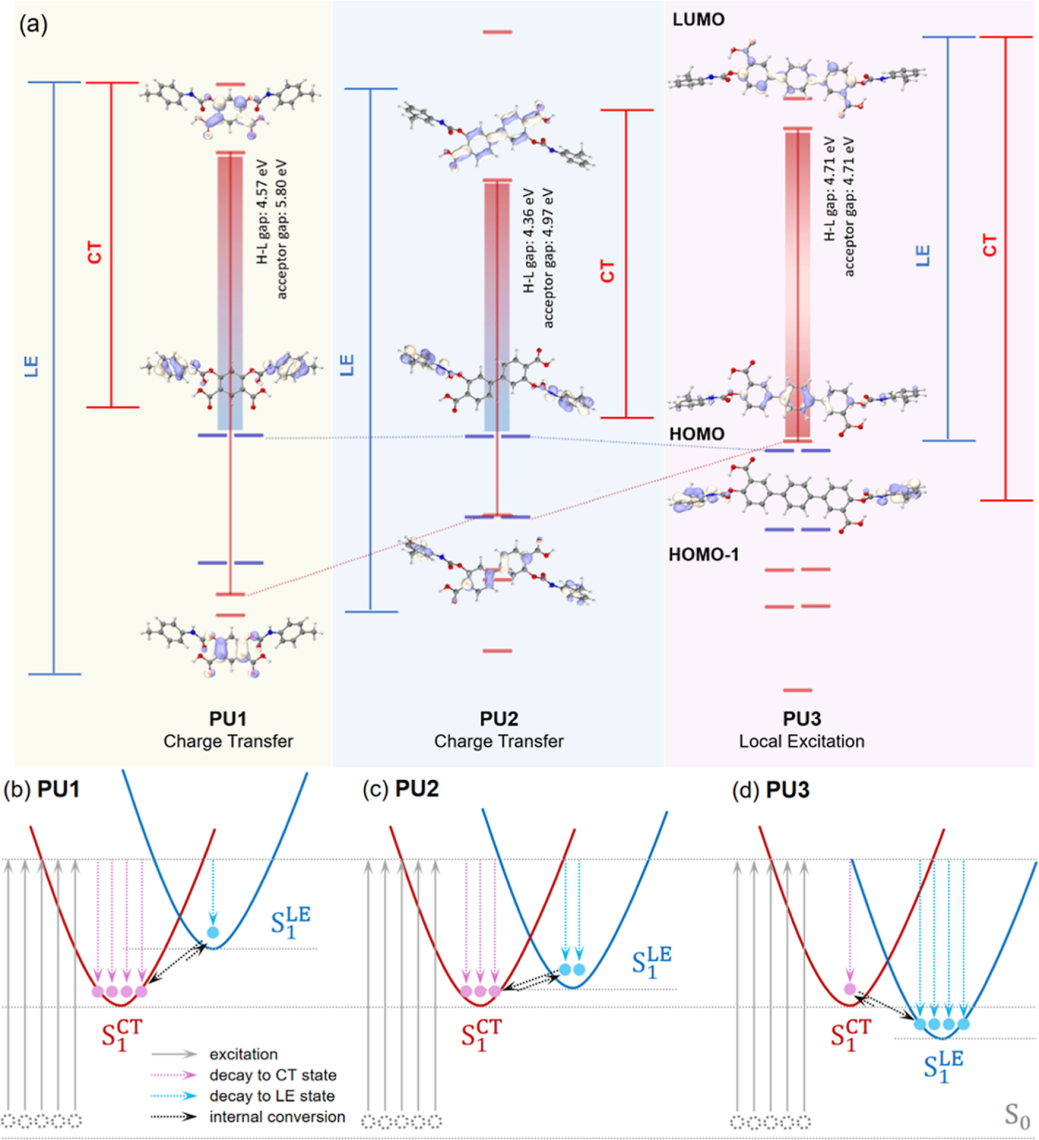
(a) π energy level crossing from **PU1** and **PU2** to **PU3** based on DFT calculations. Donor and acceptor orbitals are colored in blue and red, respectively. Schematic diagram of the excited-state potential energy surfaces and exciton population on the CT and LE states of (b) **PU1**, (c) **PU2**, and (d) **PU3**.

Being close in excitation energy (Table S4), S_1_^LE^ acts as another available excited state in **PU1** and **PU2** via direct excitation followed by fast internal conversion, while S_1_^CT^ is dominant in **PU3** instead. However, due to the difference in excited-state energies, the distribution of excitons on S_1_^CT^ and S_1_^LE^ also changes. In **PU1** and **PU2**, luminescence is controlled by the exciton population on S_1_^CT^. In the PL spectra measured with longer excitation wavelengths, peaks with mixed emission features (S_1_^CT^ and S_1_^LE^) were observed (Figure 2). Short excitation wavelength enables and increases the exciton population of the excited state with higher energy, that is, S_1_^CT^ in **PU1** and **PU2**; therefore, the S_1_^LE^ emission vanishes and pure S_1_^CT^ emission is observed in the PL spectra. The opposite phenomenon was observed for **PU3**, where S_1_^CT^ emission vanishes and S_1_^LE^ emission is enhanced with shorter wavelength excitation.

This interesting excitation-wavelength-dependent exciton population was further verified by calculating internal conversion rates between the S_1_^CT^ and S_1_^LE^ states. As listed in Table S5, for both **PU1** and **PU2**, the internal conversion rate from S_1_^CT^ to S_1_^LE^ is lower than that of its reversed process, demonstrating a larger exciton population on the S_1_^LE^ state. In contrast, **PU3** has the opposite trend (Figure S22). This can be visualized as an excited-state potential energy surface diagram (Figure 5b–d). A longer π-conjugated system leads to decreased local excitation energy in the **PUs**, as when lower excitation energy is given, PL tends to be observed only for the lowest excited state. Increasing the excitation energy (i.e., shorter excitation wavelength) gradually moves excitons to higher excited states; therefore, a mixed excitation feature is initially observed in the PL spectra and finally changes the excitation feature to CT states.

Notably, the hypsochromic (blue) shift trend (λ_max_ **PU1** > **PU2** > **PU3**) aligns with expectations: increasing phenyl rings can enhance torsional distortion and reduce conjugation, which would logically lead to a blue shift. What is surprising, however, is that **PU3** exhibits the most pronounced blue shift alongside a higher quantum yield. This observation, where quantum yield does not decrease with more phenyls, demands clarification through structural relaxation and conformational dynamics. **PU1** contains a single phenyl and does not have a directly linked biphenyl structure, whereas **PU2** contains two (forming a biphenyl) and **PU3** three (forming a terphenyl), with these phenyl groups linked via conjugated backbones. Increasing phenyls rigidify π-moieties, elevating the torsional force constants (*k*_torsion_). For extended π-systems, potential energy (*V*) depends strongly on dihedral angles: *V*(θ) ∝ *k*_torsion_·θ2, reducing vibrational nonradiative relaxation. In polymer matrices, the surrounding chains further restrict torsion (e.g., **PU3**’s terphenyl resists distortion more than **PU1**’s biphenyl), lowering reorganization energy (λ ∝ Δ*q* ^2^/*k*_torsion_). This suppresses nonradiative decay, which specifically explains why **PU3** maintains higher quantum yield despite its enhanced torsional distortion (Table S1). Multiphenyl extension creates rigid π-domains, rationalizing the hypsochromic trend via stiffened torsion and matrix constraints, distinct from simple R–H/R–Ph substitutions.^41^

### 2.4 Applications

Given that the **PUs** exhibit different colors under ambient room light or 365 nm UV light, they were selected as assembly modules to verify their application potential in information transmission and colorful displays (Figures 6 and S23). As shown in Figure 6b, the **PUs** were used for the pixel painting of a dog holding a heart. Under room light, the painting appears tan and dark-yellow. However, under a 365 nm UV light, a bright blue dog is obtained with a luminous yellow heart and a dark yellow-green background. Additionally, to explore the practical application value of the aggregation-induced emission of the **PUs**, **PU1** was doped into poly­(methyl methacrylate) (PMMA) in DMF solutions, and then **PU1**-PMMA films were prepared by evaporation (Figure 6c). The amount of PMMA was fixed at 500 mg, while the amount of **PU1** was varied: plastic films with progressive blue to green fluorescence were obtained. The emission spectra and photographs are shown in Figure 6c. Due to limited solubility, membranes with a higher proportion of **PU1** were not prepared.

**Figure 6:**
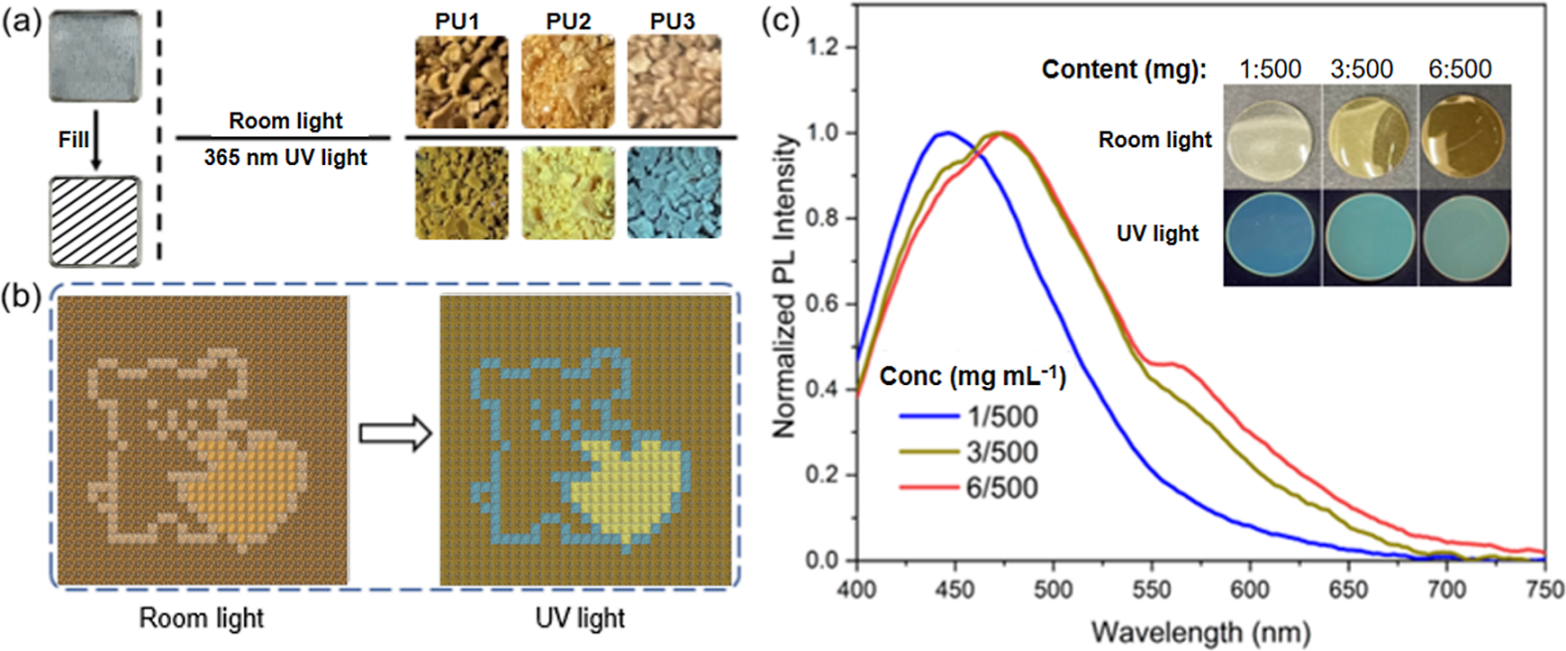
(a) Photographs of **PU1**, **PU2**, and **PU3** in room light and UV light. (b) The pixel painting and its photograph under room light or UV light, respectively. (c) Normalized emission spectra of **PU1**–PMMA films with different doping ratios, and the corresponding photographs of the films under room light or UV light.

## 3 Conclusion

Three **PU** derivatives (**PU1–3**) with varying degrees of separation of aromatic rings were synthesized to provide insights into the modulation of the electronic structure in **NCLPs**. The **PUs** show intrinsic emission from benzene rings in dilute solution but different photophysical properties in the solid (aggregate) state. The concentration-dependent emission spectra reveal that the peak positions of **PU1** and **PU2** solutions remain nearly unchanged with increasing concentration, whereas **PU3** exhibits continuous red-shifting and broadening of the emission peaks at higher concentrations, ascribed to the existence of complex spatial electronic conjugation processes in the aggregated state. DFT calculations and MD simulations demonstrate that hydrogen bonds and *p*–π* interactions regulate emission wavelengths by modulating aggregated-state structures. Furthermore, an increase in benzene ring numbers (benzene, biphenyl, and terphenyl) reduces the energy gap of the acceptor moieties, and these synergistic effects lead to distinct PL profiles in the **PUs**. Benefiting from the excellent aggregation-state-dependent PL properties, the **PUs** have been successfully applied in dual-mode display patterns and tunable fluorescent film fabrication. Overall, this work advances the exploration of structure–activity relationships, the underlying excitation-dependent properties, and the multifunctional applications of nonconventional luminescent materials, providing a versatile strategy for regulating the electronic structure and photophysical properties.

## Supplementary Material



## Data Availability

The data associated with this article is available in the manuscript and Supporting Information files.

## Notes

Additional structural characterization; copies of NMR spectra; FT-IR spectra; additional photophysical data; cyclic voltammetry data; scanning electron microscopy (SEM) images; and computational data (PDF)
