# Supplementary material for: Non-Traditional Luminescent Polyurethanes of n–π Electron Hybrid Structures with Varying Separation of Aromatic Rings
Source: ACS Appl Polym Mater. 2025 Sep 12;7(18):12337–44. doi: 10.1021/acsapm.5c02091 (PMC12481474; doi:10.1021/acsapm.5c02091)
Supplement: Supplementary file 1 [file ap5c02091_si_001.pdf]

Supporting Information

Non-Traditional Luminescent Polyurethanes of n-π Electron Hybrid Structures with Varying Separation of Aromatic Rings

Ziwei Wang,†[a,c] Yingqi Li,†[b] Han Zhang,[a] Nan Jiang,*[a] Jiawei Xu,*[b] Dongxia Zhu[c] and Martin R. Bryce*[d]

[a] Key Laboratory of Preparation and Applications of Environmental Friendly Materials (Jilin Normal University), Changchun, 130103, China.

[b] Ministry-of-Education Key Laboratory of Numerical Simulation of Large-Scale Complex System (NSLSCS) and School of Chemistry and Materials Science, Nanjing Normal University, Nanjing 210023, China.

[c] Key Laboratory of Nanobiosensing and Nanobioanalysis at Universities of Jilin Province, Faculty of Chemistry, Northeast Normal University, Changchun 130024, China

[d] Department of Chemistry, Durham University, Durham, DH1 3LE, UK

E-mails: jiangn270@jlnu.edu.cn; jwxu_njnu@sina.com; m.r.bryce@durham.ac.uk

Contents:

1. Experimental details

2. Structural characterization

3. Photophysical properties

4. Theoretical calculations

5. References

1. Experimental details

General

The UV-vis absorption spectra were recorded on a Shimadzu UV-3100 spectrophotometer. ^1H NMR spectra were recorded on a Bruker AVANCE III HD 400. The ^1H NMR spectra were referenced internally to the residual proton resonance in DMSO-d_6 (δ 2.5 ppm). The molecular weights of the polyurethane were determined by gel permeation chromatography (GPC) on a PL-GPC120 instrument with polystyrene as the reference and DMF as the eluent at 80 °C. Scanning electron microscope (SEM) images were obtained using a JEOL model JSM-6700 instrument operating at an accelerating voltage of 5.0 kV. The samples were prepared by placing microdrops of the solution on a holey carbon copper grid. The luminescence photographs were taken by an iPhone 14 pro under the irradiation of a hand-held UV lamp at room temperature. Photoluminescence spectra and fluorescence lifetime were measured using an Edinburgh FS5 instrument. Fluorescence quantum efficiency was determined using an Edinburgh Instruments FS5 spectrofluorometer equipped with an integrating sphere, with reference to a calibrated solid reference standard. The quantum yield was calculated as the ratio of the number of emitted photons to the number of absorbed photons, and the data were processed using FS5 software. The fluorescence microscope images were obtained using a ZEISS Scope.A1 with HBO 100 illuminator. The ZEISS Scope.A1 was equipped with three fluorescence modules. When Ex=365 nm, Em collects light >420 nm; when Ex=400-460 nm, Em=500-600 nm; when Ex=420-450 nm, Em collects light >515 nm.

Synthesis of PU1

A mixture of 4,6-dihydroxybenzene-1,3-dicarboxylic acid (0.208 g, 1.048 mmol), anhydrous THF (3 mL), anhydrous DMSO (2 mL), 2,4-toluenediisocyanate (0.274 g, 1.572 mmol) and 1,4-diazabicyclo[2,2,2]octane (DABCO) (0.047 g, 0.042 mmol) were added to a dried two-neck round-bottom flask. The solution was heated at 75°C for 24 h under nitrogen atmosphere. After that time the clear solution became significantly viscous, indicating the polymerization reaction had occurred. The crude product was dissolved in chloroform and then reverse

precipitated from excess diethyl ether. Then the product was dried under vacuum for 24 h to obtain the resulting **PU1** (0.241 g); yield 50%.

Synthesis of PU2

A mixture of 3,3-dihydroxy-[1,1-biphenyl]-4,4-dicarboxylic acid (0.216 g, 0.786 mmol), anhydrous THF (3 mL), anhydrous DMSO (3 mL), 2,4-toluenediisocyanate (0.205 g, 1.179 mmol) and DABCO (0.004 g, 0.032 mmol) were added to a dried two-neck round-bottom flask. The solution was heated at 75°C for 24 h under nitrogen atmosphere. After that time the clear solution became significantly viscous, indicating the polymerization reaction had occurred. The crude product was dissolved in chloroform and then reverse precipitated from excess diethyl ether. Then the product was dried under vacuum for 24 h to obtain the resulting **PU2** (0.242 g); yield 58%.

Synthesis of PU3

A mixture of 4,4''-dihydroxy-[1,1':4',1''-terphenyl]-3,3''-dicarboxylic acid (0.184 g, 0.524 mmol), anhydrous THF (5 mL), anhydrous DMSO (7 mL), 2,4-toluenediisocyanate (0.137 g, 0.786 mmol) and DABCO (0.002 g, 0.021 mmol) were added to a dried two-neck round-bottom flask. The solution was heated at 75°C for 24 h under nitrogen atmosphere. After that time the clear solution became significantly viscous, indicating the polymerization reaction had occurred. The crude product was dissolved in chloroform and then reverse precipitated from excess diethyl ether. Then the product was dried under vacuum for 24 h to obtain the resulting **PU3** (0.204 g); yield 64%.

SEM sample preparation

Different concentrations of PUs/DMSO solutions (0.1-20 mg mL^{-1}) were prepared and transferred into 5 mL centrifuge tubes. The centrifuge tubes were shaken thoroughly on a vortex mixer until the samples were completely dissolved. Shaking of the centrifuge tubes was continued for another 30 minutes. The angle and force of the centrifuge tube was adjusted every 10 minutes to ensure that the samples were fully dispersed in the DMSO solution. 1 mL of the prepared PUs/DMSO solution was dispersed in 1 mL of ethanol. Continued use of the vortex

mixer dispersed the sample and prevented PU nanoparticles from aggregating in the solution. The samples were dropped onto a holey carbon copper grid, the solvent was evaporated overnight, and finally, the SEM instrument was used to observe the nanoaggregate structure caused by the evaporation of the solvent.

Theoretical calculations

The initial models of three systems **PU1**, **PU2** and **PU3** were built by Packmol program and 50 model molecules of **PU1** or **PU2**/**PU3** (m = 2 and n = 2) were placed in the orthogonal box. The model molecular **PU1** or **PU2**/**PU3** was optimized at the PBE0-D3(BJ)/def2-SVP level of theory[1-4] using Gaussian 16 (Revision C.01)[5] and no imaginary frequency was checked by frequency calculation. The restrained electrostatic potential (RESP) atomic charges were generated by Multiwfn.[6] Force field parameters were adopted from generalized Amber force field (GAFF).[7] TD-DFT calculations were performed at the CAM-B3LYP-D3/def2-SVP level of theory based on optimized structures of the photofunctional unit for **PU1**, **PU2** and **PU3**. In total 10 lowest excited states were solved in TD-DFT iterations to obtain an accurate excitation energy of the S_1 state.

Molecular dynamics (MD) simulations were performed using the GROMACS (version 2024.3) package[8] and topology file and forcefield parameters were created by Sobtop.[9] The long-range electrostatic interactions were handled by the particle-mesh Ewald (PME) method and the cutoff value of van der Waals interactions was set to 10.0 Å.[10] After energy minimization, the three systems were heated up from 0 K to 300 K in the 1.0 ns simulations. Subsequently, the 50.0 ns MD simulations (MD1) were conducted in the NPT ensemble at 300 K using the v-rescale thermostat method[11] and the Berendsen method.[12] Next, the three systems were heated up to synthetic temperatures 348 K, with the 50.0 ns of MD simulations (MD2). Finally, the temperature of the three systems dropped to 300 K and unrestrained MD simulations for 50.0 ns (MD3) were performed.

For the internal conversion rates, we employed the time-independent Fermi-Golden rule approach (eq. 1) with derivative coupling as the perturbation (eq. 2). The rate expression implemented in FCClasses 3 program is eq. 3.

$$k_{\mathrm{nr}}=\frac{2\pi}{\hbar}\sum_{v_i}\sum_{v_f}\rho_{v_i}(T)\ \left|\left\langle v_i\left|\widehat{H}_{if}^{'}\right|v_f\right\rangle\right|^2\ \delta\left(\hbar\omega_f\text{-}\hbar\omega_i+\Delta E\right) \quad \text{eq.1}$$

$$\widehat{H}_{if}^{'}=\text{-}\hbar^{2\,\Sigma_k\Lambda_{if,k}}\ \frac{\partial}{\partial Q_k} \quad \text{eq.2}$$

$$k_{\mathrm{IC}}=\frac{2\pi}{\hbar^3}\sum_{v_i}\sum_{v_f}\rho_{v_i}(T)\ \left|\left\langle v_i\middle|\Lambda_{if}!\cdot!\nabla_Q\middle|v_f\right\rangle\right|^2\delta\left(\hbar\omega_f\text{-}\hbar\omega_i+\Delta E\right) \quad \text{eq.3}$$

2. Structural characterization

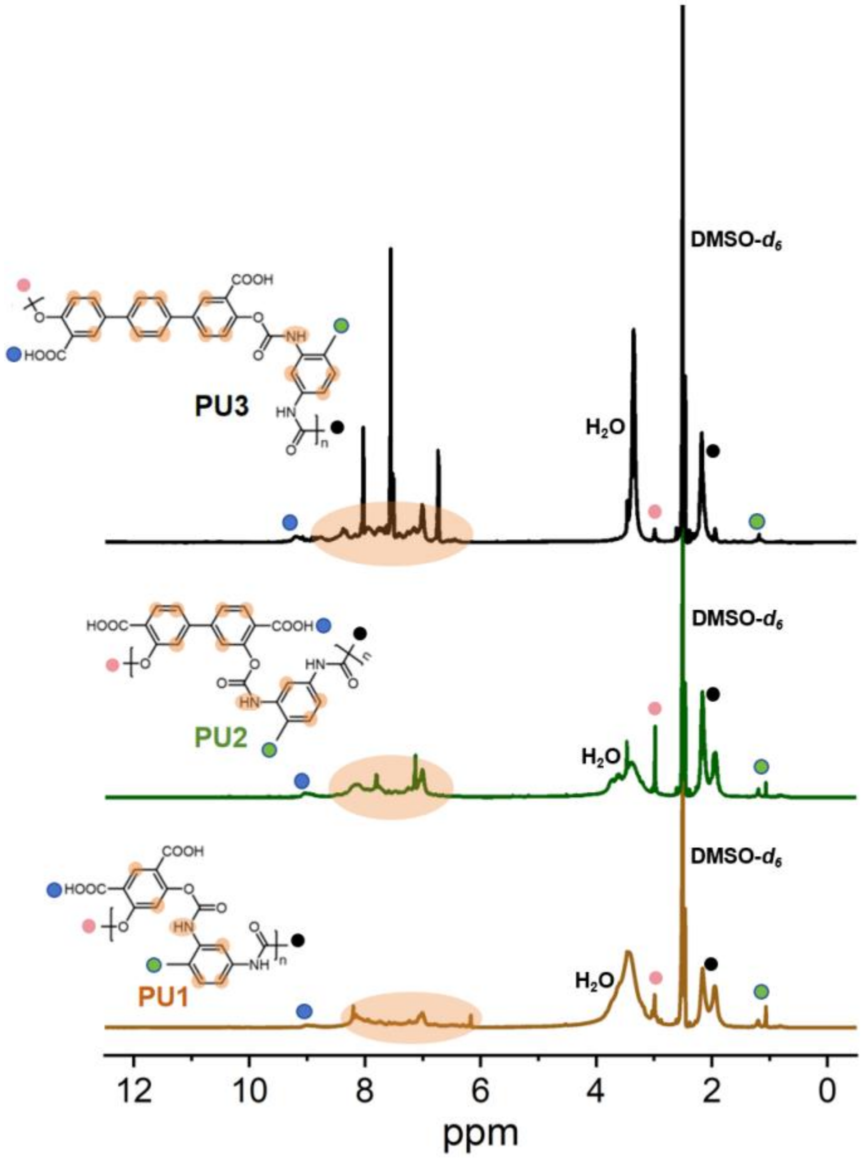

Figure S1. ^{1}H NMR spectrum of the PUs in DMSO-d_6.

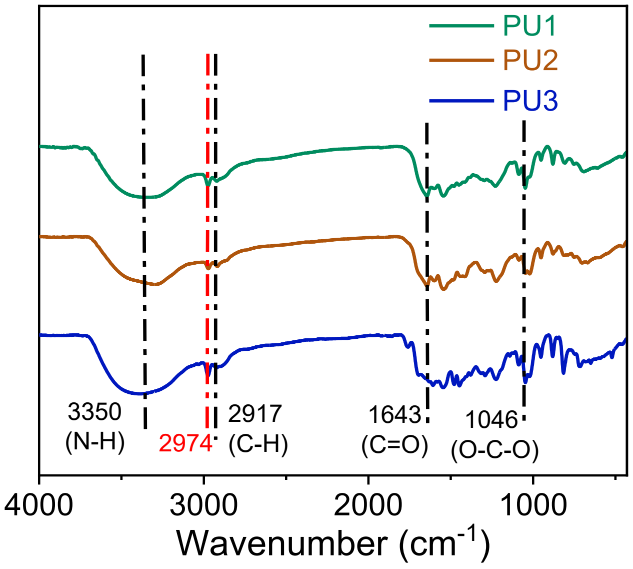

Figure S2. FTIR spectra of the PUs in the solid state.

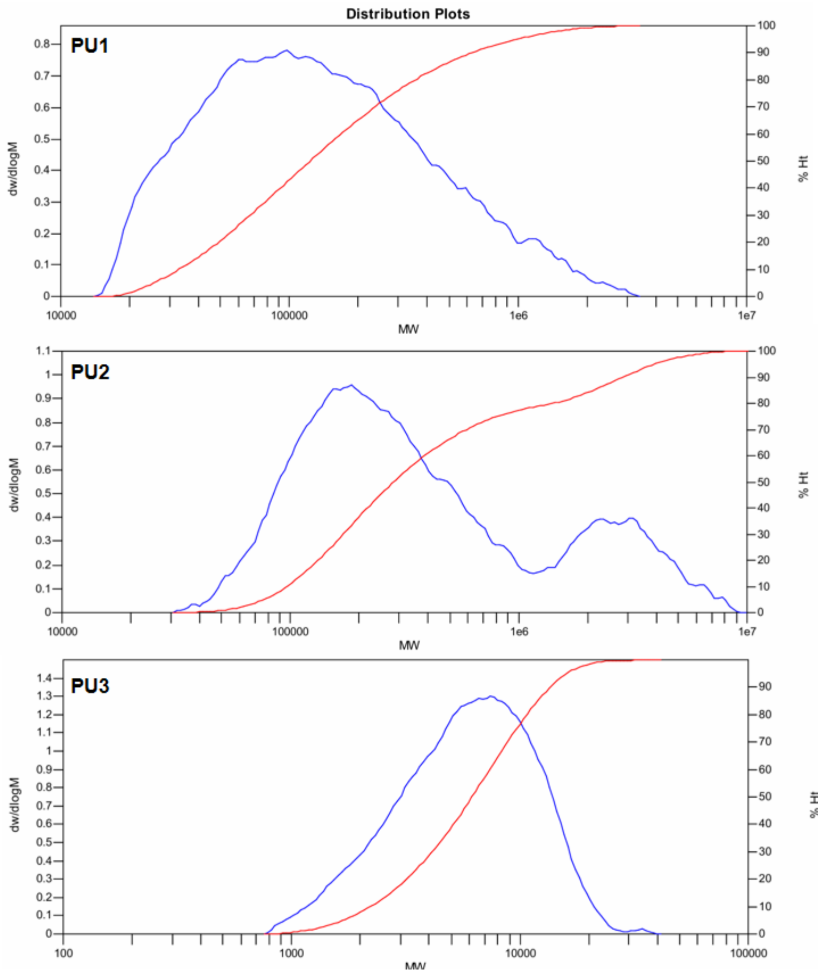

Figure S3. GPC molecular weight distribution plots of solid **PU1**, **PU2**, and **PU3.**

3. Photophysical properties and micromorphology

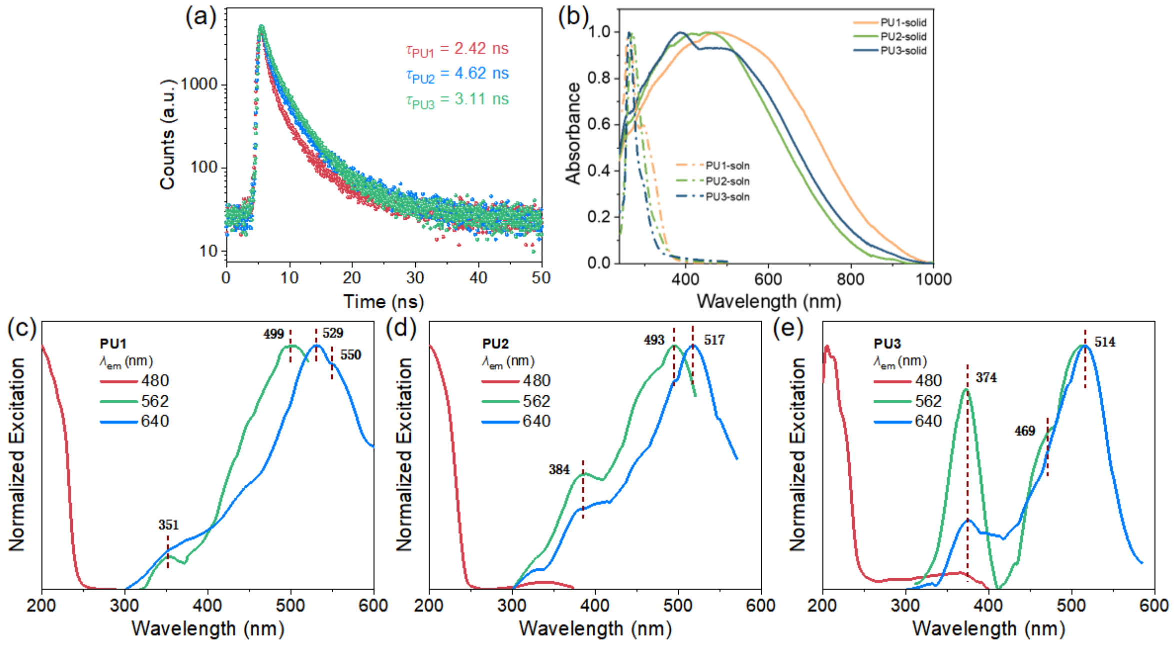

Figure S4. (a) Fluorescence photoluminescence lifetime of solid **PU1**, **PU2** and **PU3**. (b) Normalized UV-vis absorption spectra of **PU1**, **PU2** and **PU3** in the solid state and in 0.1 mg mL^{-1} DMSO solvent. Excitation spectra of solid-state (c) **PU1**, (d) **PU2**, and (e)**PU3**, monitored at emission peaks of 480 nm, 562 nm, and 640 nm, respectively.

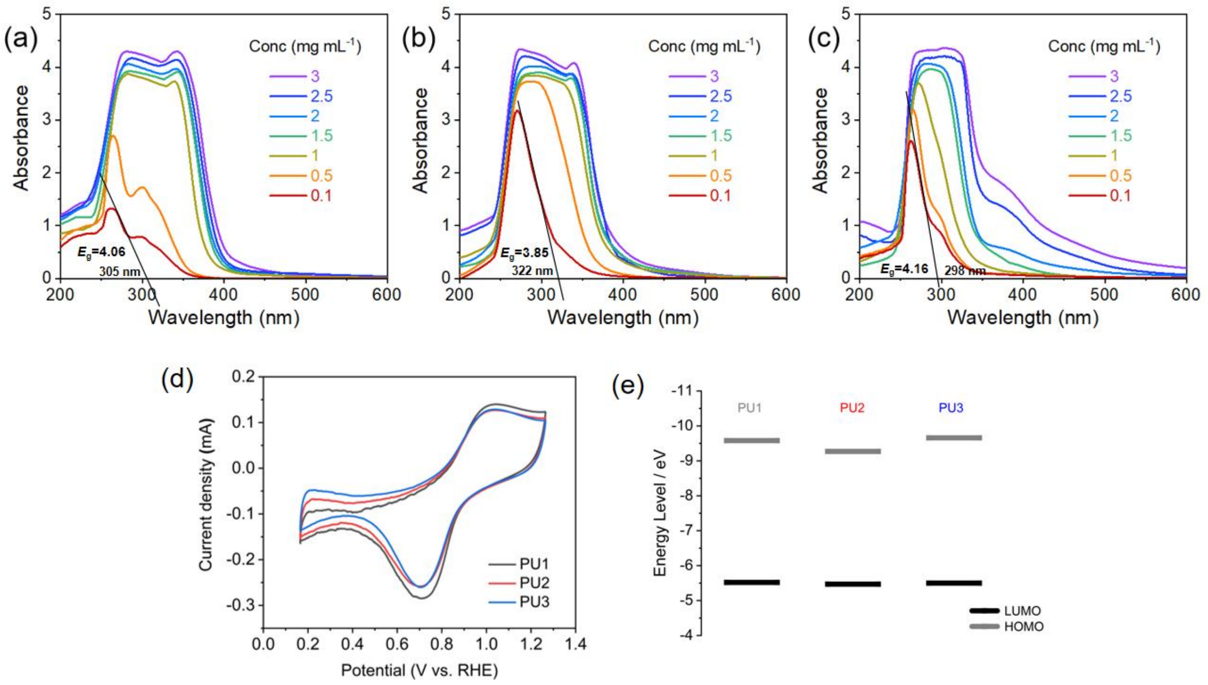

Figure S5. UV-vis absorption spectra of (a) **PU1**, (b) **PU2** and (c) **PU3** in DMSO solution at different concentrations. (d) Cyclic voltammetry plots of **PU1**, **PU2**, and **PU3**, Fc (ferrocene) as external standard, 0.1 M Na_2CO_3 as electrolyte, scan rate 100 mV s^{-1}. (e) Comparison of the experimentally determined energy levels of **PU1**, **PU2**, and **PU3**. LUMO levels are

determined as E=−(4.8+E_{red}) eV and HOMO levels as E_{HOMO}=E_{LUMO}−E_g using energy gaps (E_g) from UV-vis spectroscopy according to the equation E_g = 1240/λ_{abs}.

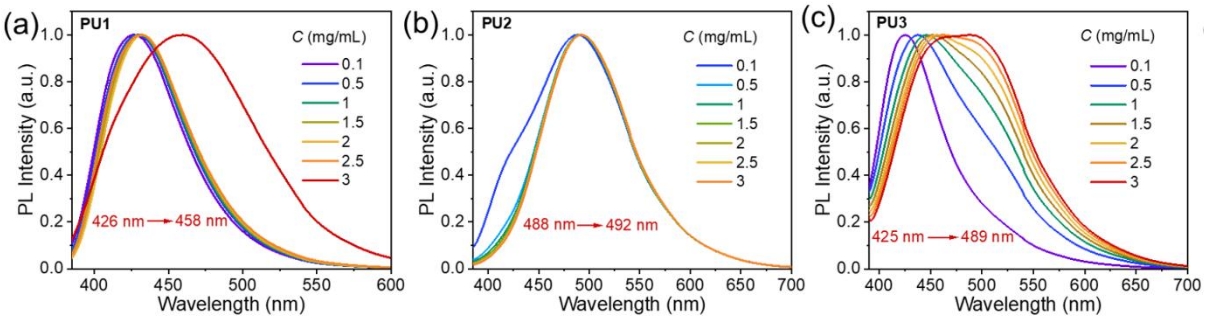

Figure S6. Normalized PL spectra of (a) **PU1**, (b) **PU2**, and (c) **PU3** in DMSO solvent at different concentrations.

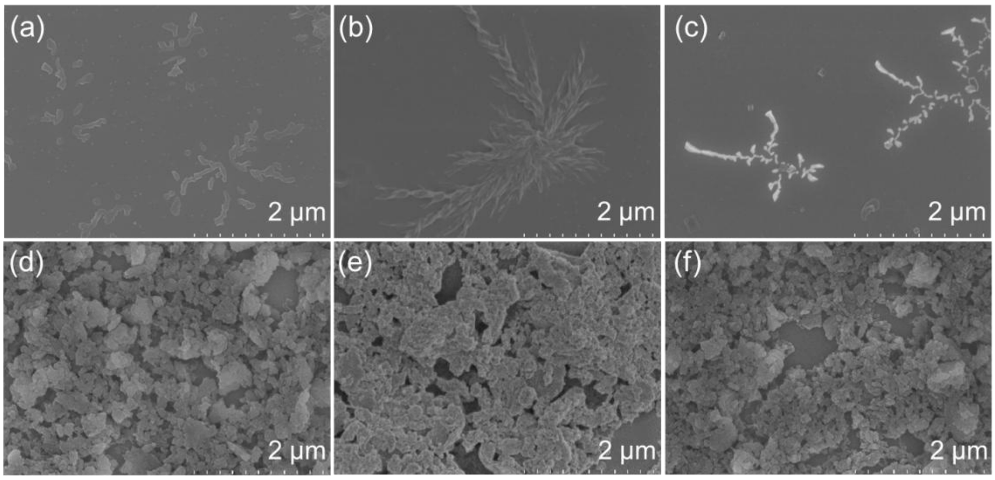

Figure S7. SEM images of 20 mg mL^{-1} (a) 4,6-dihydroxyisophthalic acid, (b) 3,3'-dihydroxy-[1,1'-biphenyl]-4,4'-dicarboxylic acid, and (c) 4,4''-dihydroxy-[1,1':4',1''-terphenyl]-3,3''-dicarboxylic acid monomer dispersed in ethanol. SEM images of 20 mg mL^{-1} (d) **PU1**, (e) **PU2**, (f) **PU3** dispersed in ethanol.

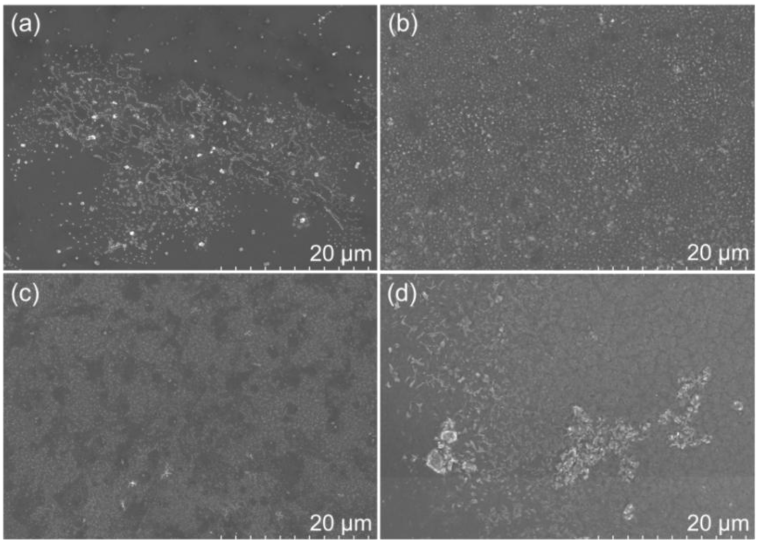

Figure S8. SEM images of (a) 0.1 mg mL^{-1}, (b) 1 mg mL^{-1}, (c) 2 mg mL^{-1}, (d) 3 mg mL^{-1} of **PU1** dispersed in ethanol.

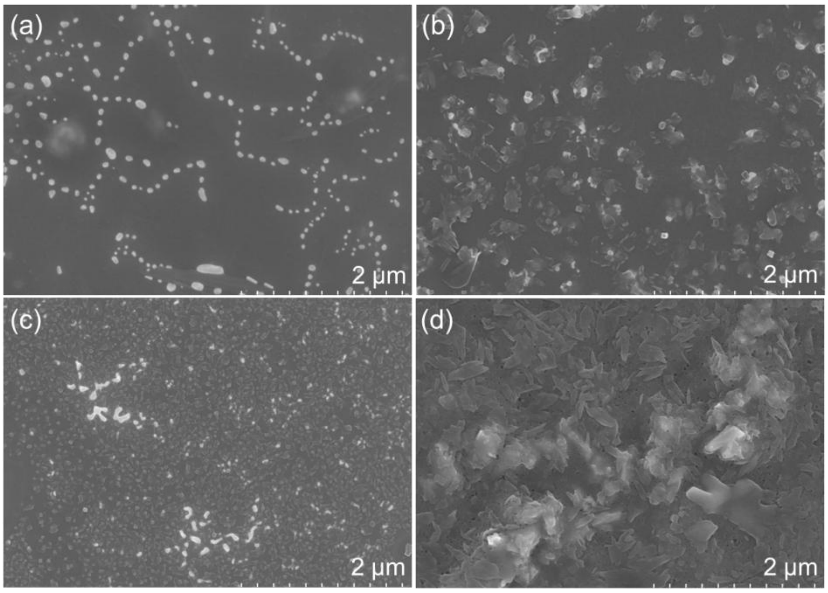

Figure S9. SEM images of **PU1** (a) 0.1 mg mL^{-1}, (b) 1 mg mL^{-1}, (c) 2 mg mL^{-1}, and (d) 3 mg mL^{-1} in DMSO solution.

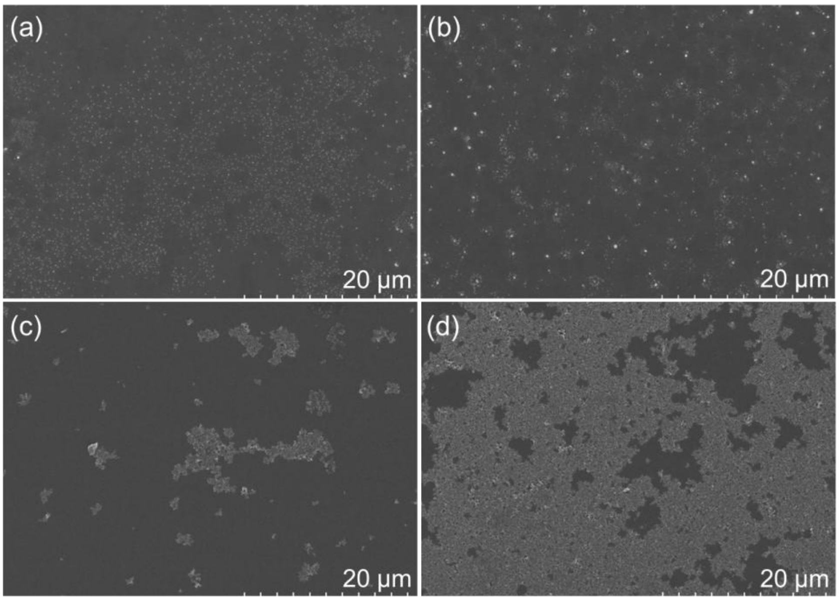

Figure S10. SEM images of (a) 0.1 mg mL^{-1}, (b) 1 mg mL^{-1}, (c) 2 mg mL^{-1}, (d) 3 mg mL^{-1} of **PU2** dispersed in ethanol.

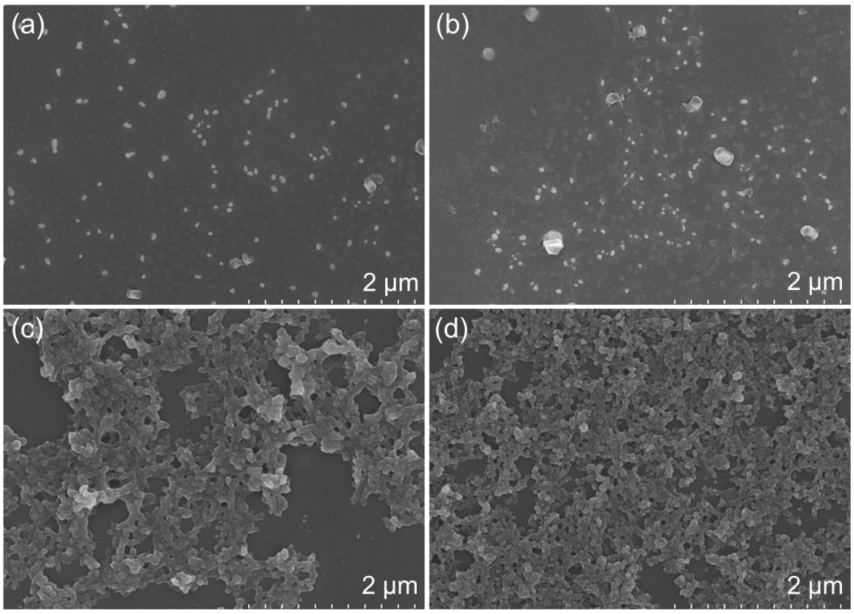

Figure S11. SEM images of **PU2** (a) 0.1 mg mL^{-1}, (b) 1 mg mL^{-1}, (c) 2 mg mL^{-1}, and (d) 3 mg mL^{-1} in DMSO solution.

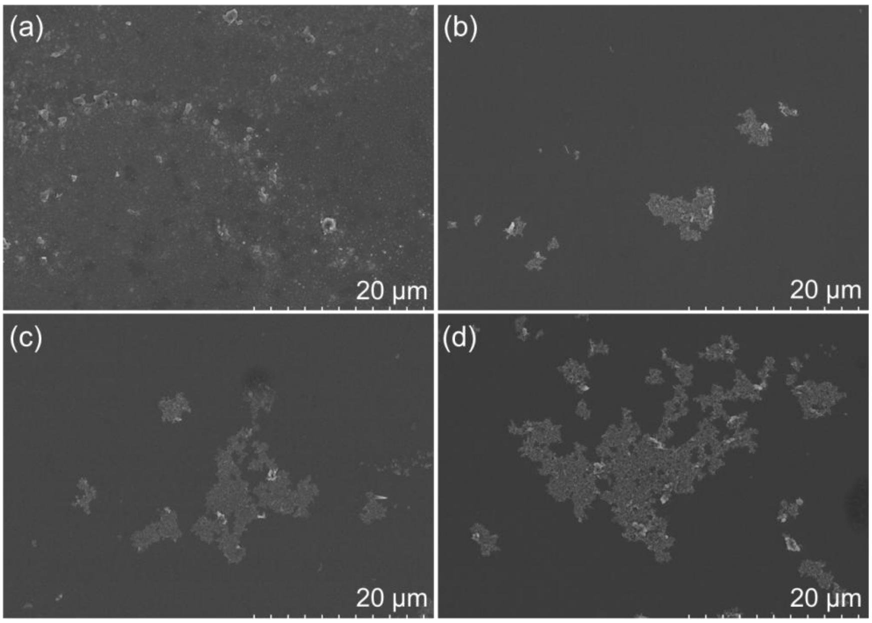

Figure S12. SEM images of (a) 0.1 mg mL^{-1}, (b) 1 mg mL^{-1}, (c) 2 mg mL^{-1}, (d) 3 mg mL^{-1} of **PU3** dispersed in ethanol.

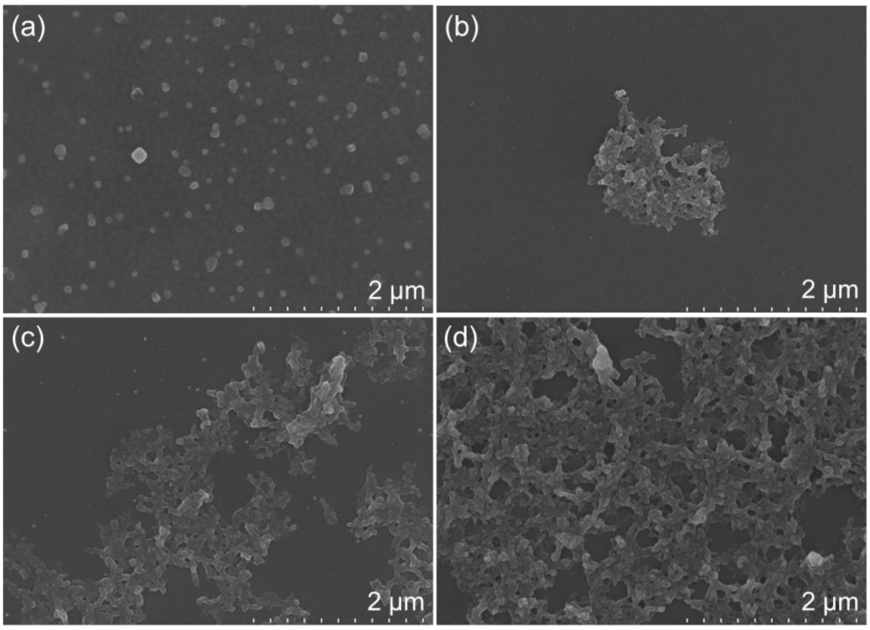

Figure S13. SEM images of **PU3** (a) 0.1 mg mL^{-1}, (b) 1 mg mL^{-1}, (c) 2 mg mL^{-1}, and (d) 3 mg mL^{-1} in DMSO solution.

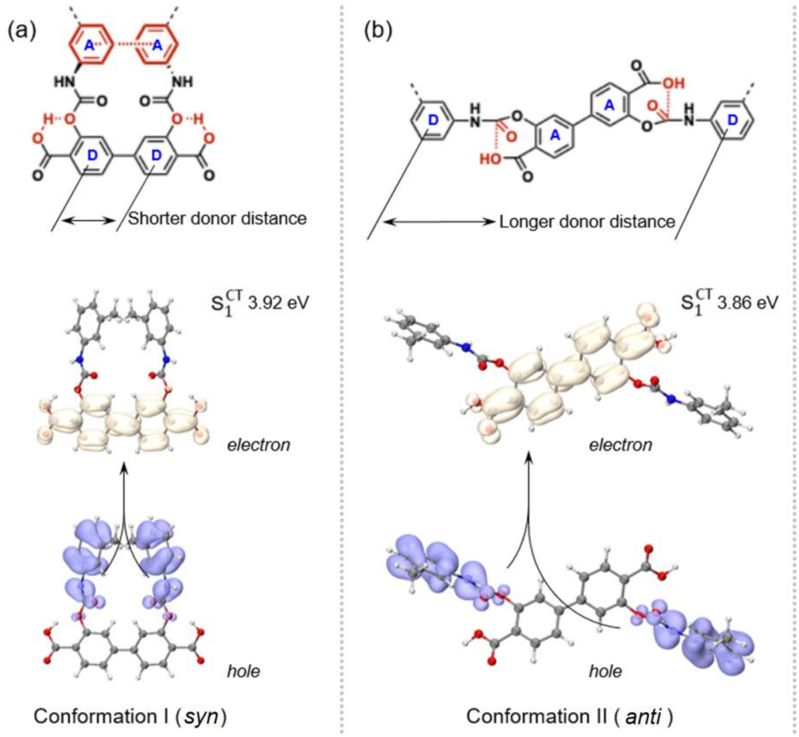

Figure S14. S_1^{CT} state electron-hole distribution of (a) *syn*- and (b) *anti*-conformation of the **PU2** photofunctional unit.

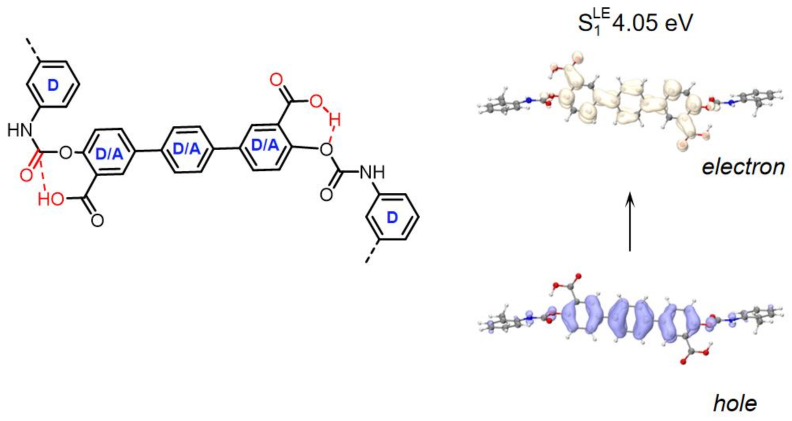

Figure S15. S_1^{LE} state electron-hole distribution of the **PU3** photofunctional unit.

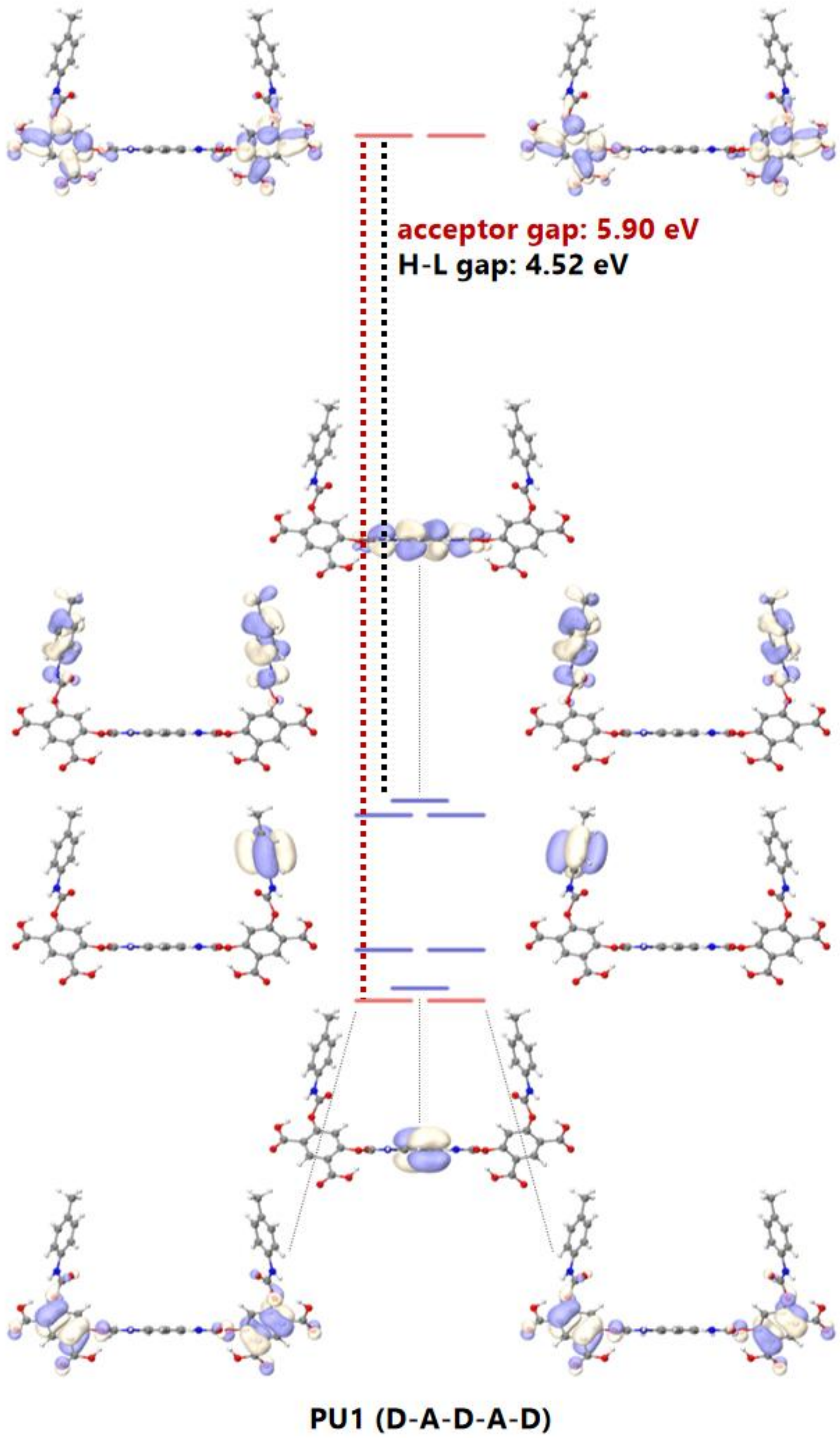

Figure S16. HOMO-LUMO gap and acceptor gap of **PU1** calculated with larger models including $(D\text{-}A)_2\text{-}D$ units.

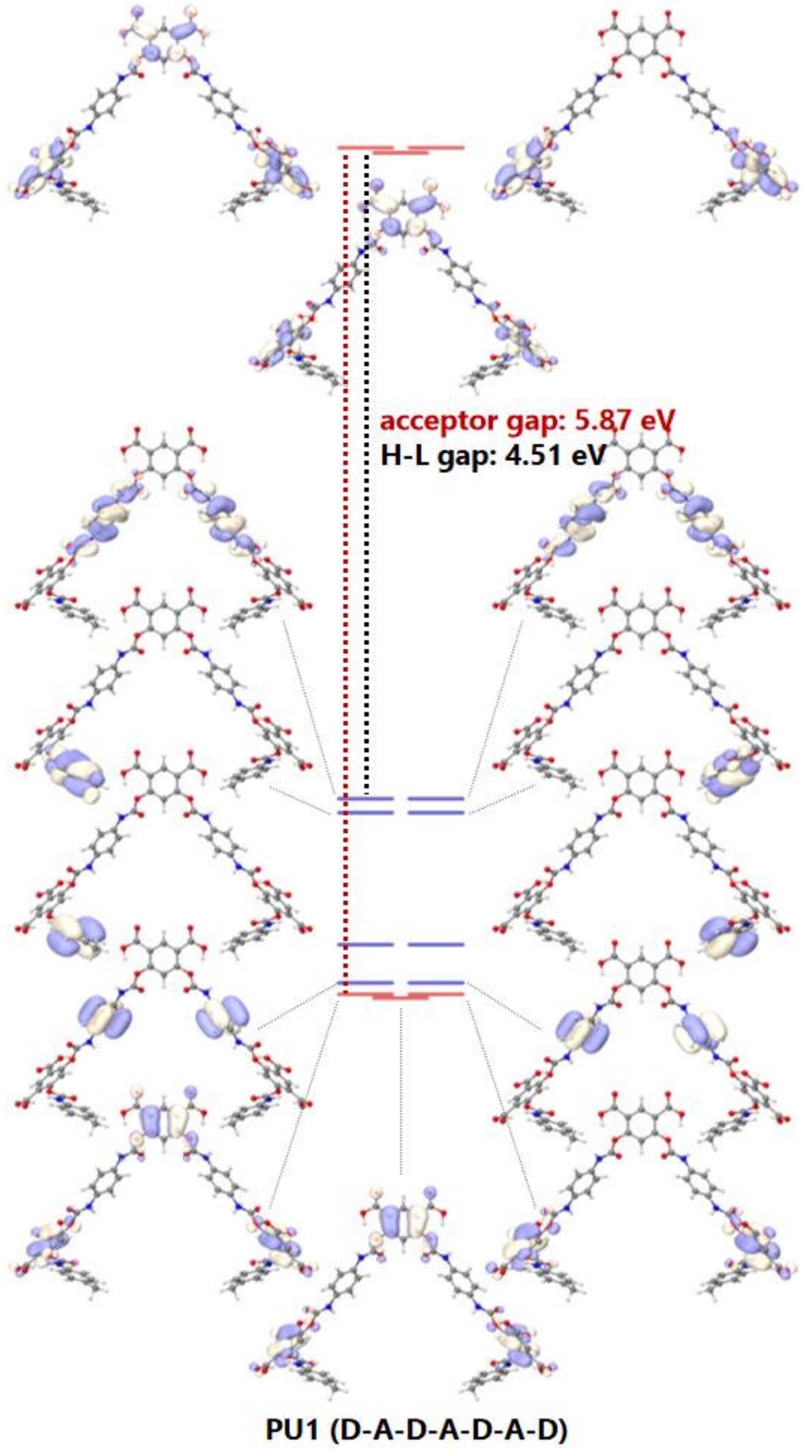

Figure S17. HOMO-LUMO gap and acceptor gap of **PU1** calculated with larger models including (D-A)$_3$-D units.

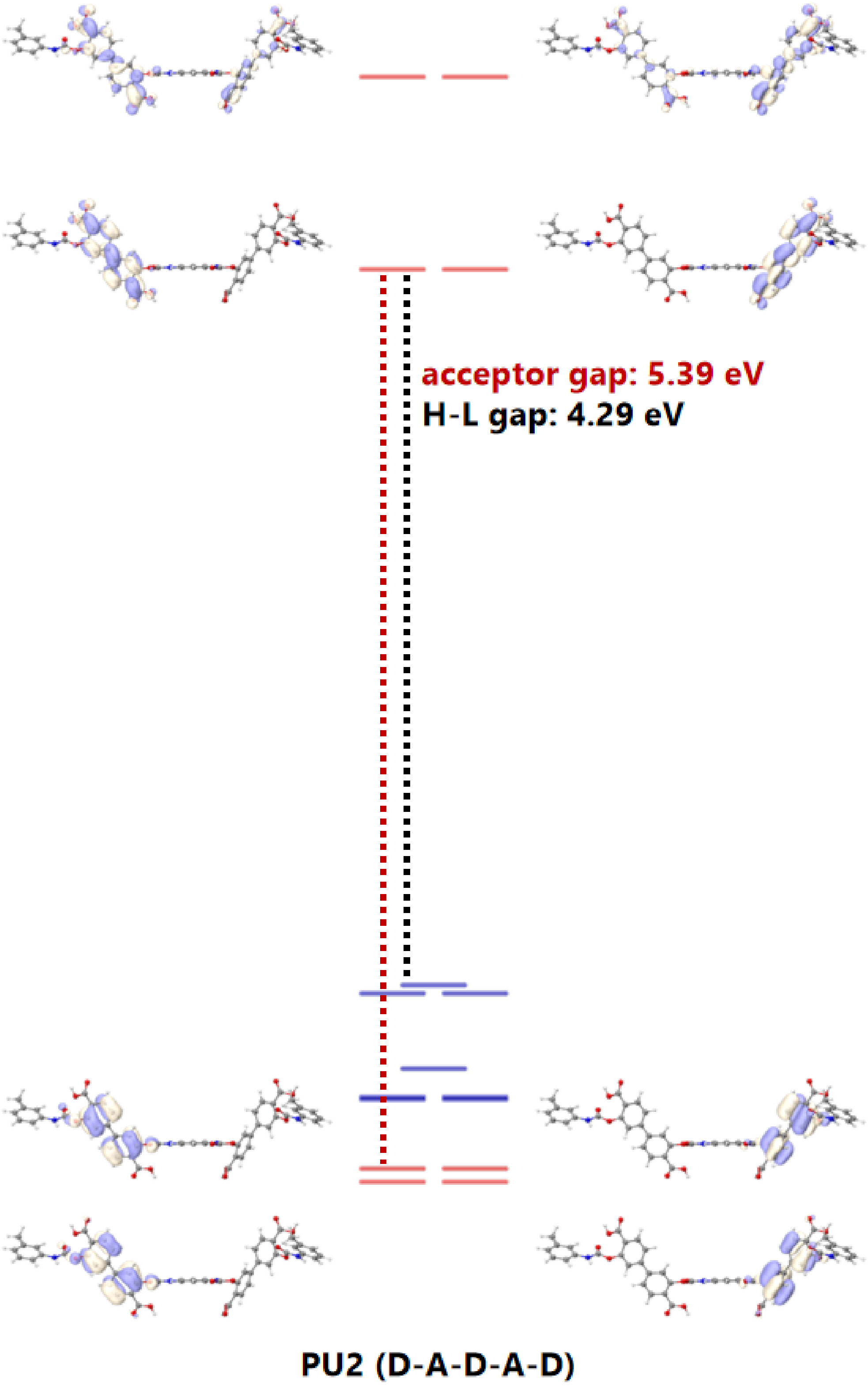

Figure S18. HOMO-LUMO gap and acceptor gap of **PU2** calculated with larger models including $(D\text{-}A)_2\text{-}D$ units.

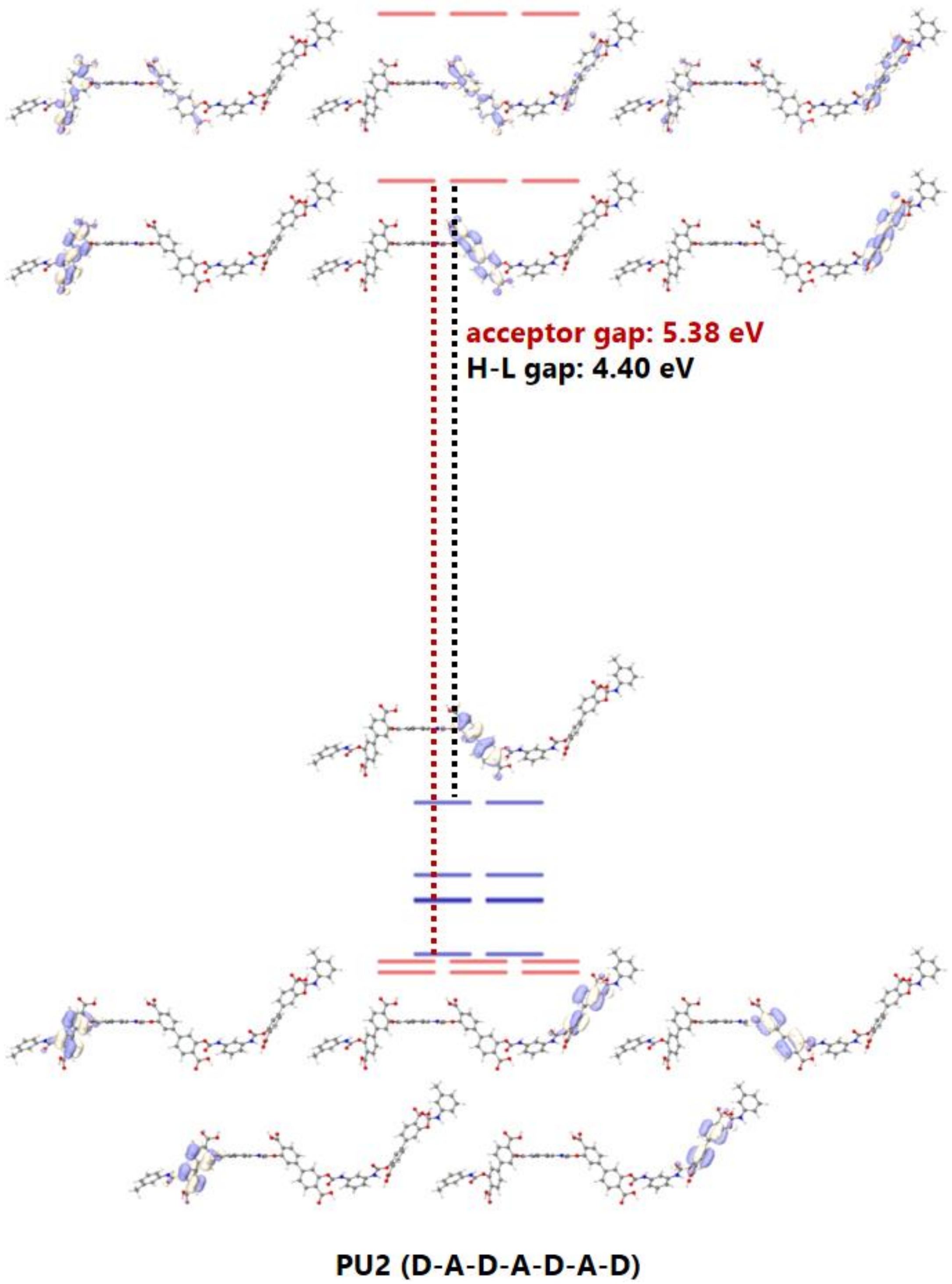

Figure S19. HOMO-LUMO gap and acceptor gap of **PU2** calculated with larger models including (D-A)$_3$-D units.

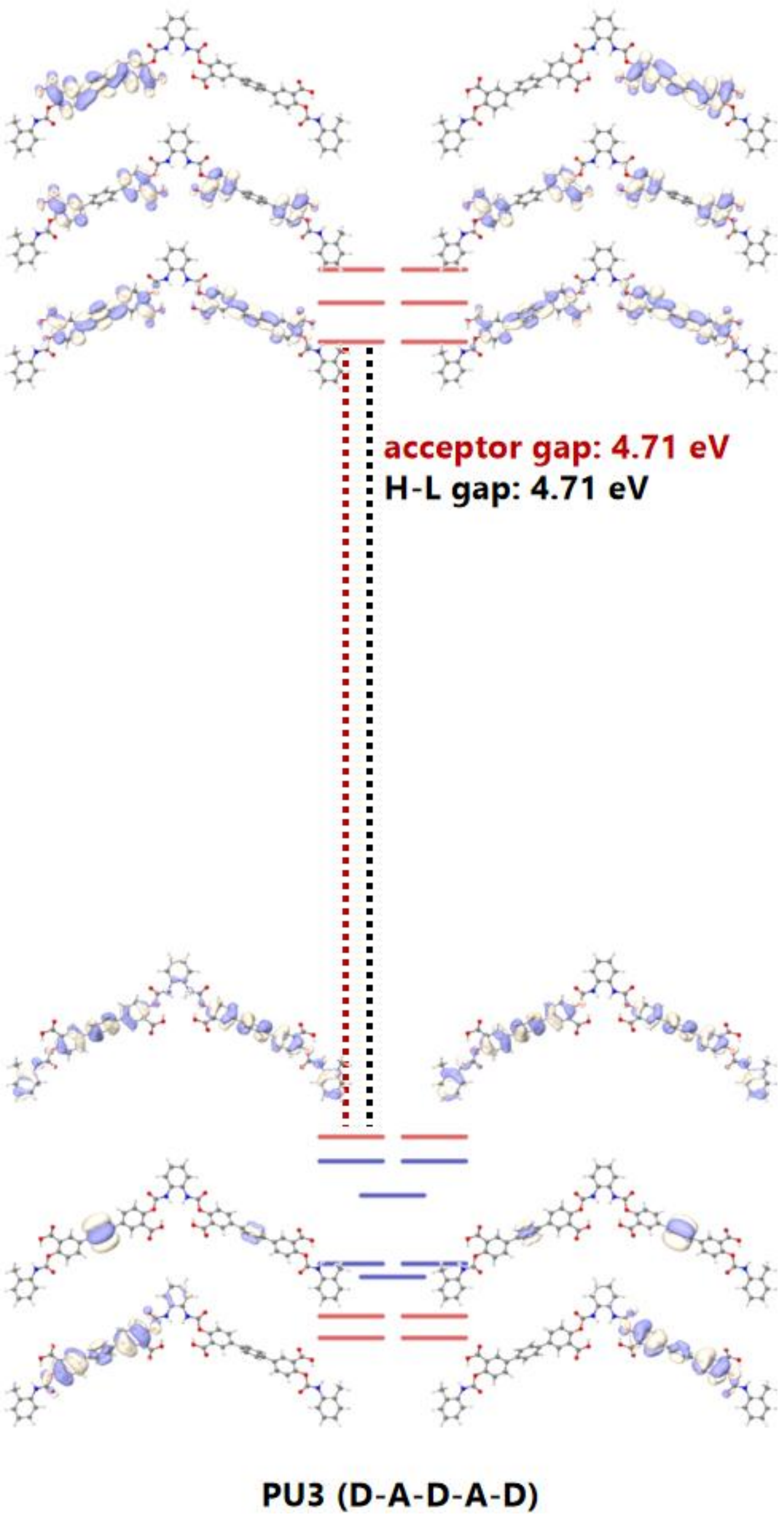

Figure S20. HOMO-LUMO gap and acceptor gap of **PU3** calculated with larger models including $(D\text{-}A)_2\text{-}D$ units.

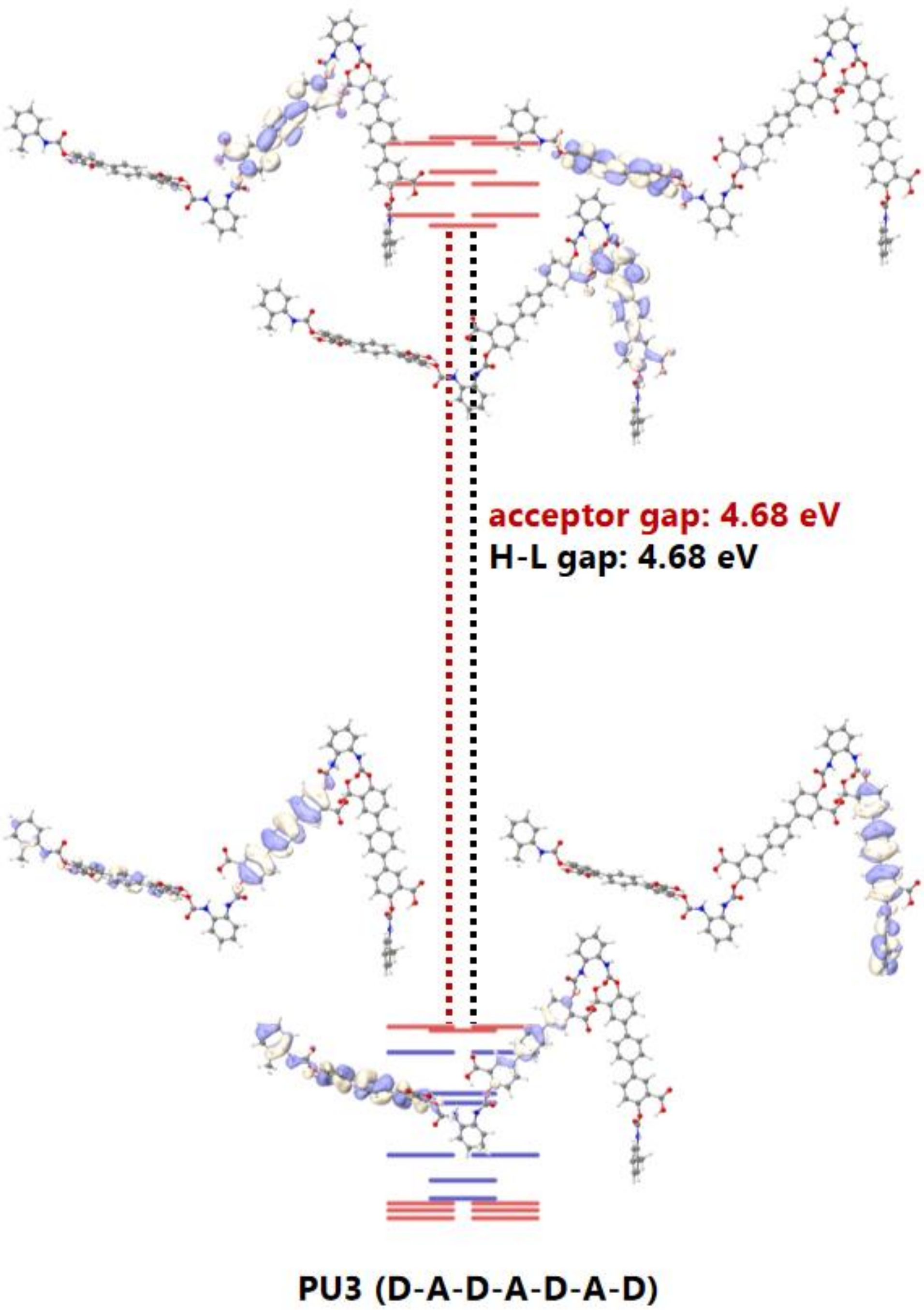

Figure S21. HOMO-LUMO gap and acceptor gap of **PU3** calculated with larger models including $(D\text{-}A)_3\text{-}D$ units.

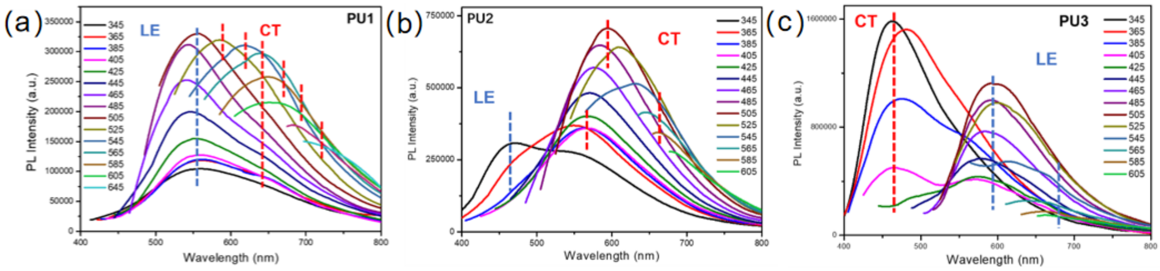

Figure S22. PL spectra of (a) **PU1**, (b) **PU2** and (c) **PU3** powders with different excitation wavelengths at room temperature.

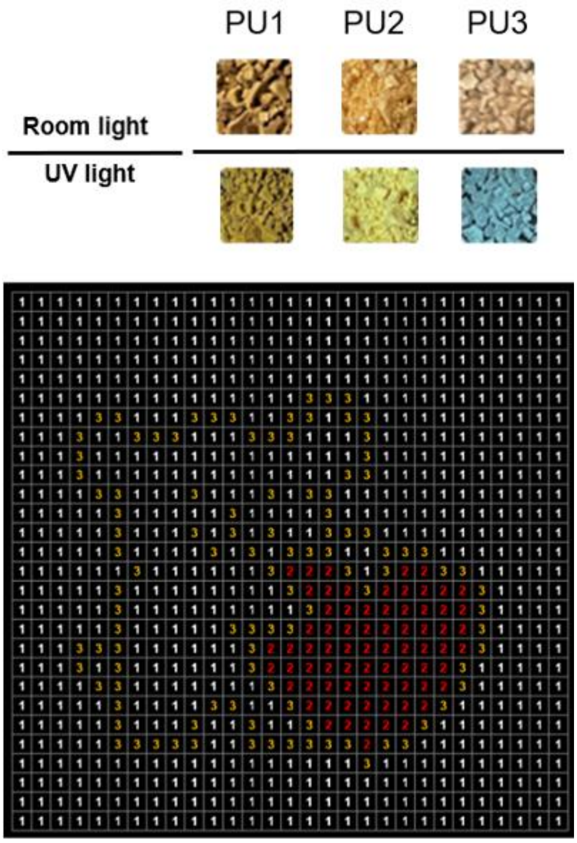

Figure S23. A schematic of the materials (**PU1**, **PU2** and **PU3**) and arrangement used in the pixel painting.

Table S1. The fluorescence quantum efficiency (QY) and luminescence lifetimes (LT) of **PU1**, **PU2** and **PU3** powder samples at room temperature.

	**PU1**	**PU2**	**PU3**
**QY**	1.7%	1.1%	2.1%
**LT(ns)[a]**	2.42	4.62	3.11
**λ_{em}(nm)[a]**	562	560	483

([a]λ_{ex}=365 nm)

Table S2. Molecular weight data of the PUs from GPC.

	M_n	M_w	M_P	M_z	**PD**
**PU1**	79261	250637	97812	749698	3.16
**PU2**	213081	874696	184723	2915010	4.10

PU3	4447	7063	7406	10038	1.59

Table S3. Average counts of hydrogen bonds in three-stage MD simulations.

	MD1 (298 K)	MD2 (348 K)	MD3 (annealed from 348 K)
**PU1**	315.70	321.10	345.19
**PU2**	315.34	323.23	342.71
**PU3**	283.64	297.67	321.16

Table S4. Excitation energy (eV) of S_1^{CT} and S_1^{LE}.

	S_1^{CT}	S_1^{LE}
**PU1**, conformation I	4.0759	4.8379
**PU1**, conformation II	3.9717	4.7967
**PU2**, conformation I	3.9212	4.3587
**PU2**, conformation II	3.8548	4.3317
**PU3**	4.3799	4.0499

Conformations for **PU1**, **PU2** and **PU3** correspond to Figures 4, S14 and S15.

Table S5. Internal conversion rate constants.

	IC[a] rate constant (s^{-1})	rIC rate constant (s^{-1})
**PU1**	4.70×10^{6}	3.14×10^{7}
**PU2**	3.14×10^{7}	6.74×10^{10}
**PU3**	1.75×10^{12}	7.37×10^{11}

[a] IC (internal conversion) denotes CT → LE process, while rIC (reversed IC) denotes LE → CT process.

4. References

1. Grimme, S. Semiempirical GGA-type density functional constructed with a long-range dispersion correction. *J. Comput. Chem.* **2006,** *27*, 1787-1799.

2. Perdew, J. P.; Burke, K.; Ernzerhof, M. Generalized Gradient Approximation Made Simple. *Phys. Rev. Lett.* **1996,** *77*, 3865-3868.

3. Papajak, E.; Leverentz, H. R.; Zheng, J.; Truhlar, D. G. Efficient Diffuse Basis Sets: cc-pVxZ+ and maug-cc-pVxZ. *J. Chem. Theory Comput.* **2009,** *5*, 3330-3330.

4. Weigend, F. Accurate Coulomb-fitting basis sets for H to Rn. *Phys. Chem. Chem. Phys.* **2006,** *8*, 1057-1065.

5. Frisch, M. J.; Trucks, G. W.; Schlegel, H. B.; Scuseria, G. E.; Robb, M. A.; Cheeseman, J. R.; Scalmani, G.; Barone, V.; Petersson, G. A.; Nakatsuji, H. et al. *Gaussian 16 Rev. C.01*, Wallingford, CT, 2016.

6. Lu, T.; Chen, F. Multiwfn: A multifunctional wavefunction analyzer. *J. Comput. Chem.* **2012,** *33*, 580-592.

7. Sprenger, K. G.; Jaeger, V. W.; Pfaendtner, J. The General AMBER Force Field (GAFF) Can Accurately Predict Thermodynamic and Transport Properties of Many Ionic Liquids. *J. Phys. Chem. B.* **2015,** *119*, 5882-5895.

8. Hess, B.; Kutzner, C.; Van Der Spoel, D.; Lindahl, E. GROMACS 4: algorithms for highly efficient, load-balanced, and scalable molecular simulation. *J. Chem. Theory Comput.* **2008,** *4*, 435-447.

9. Tian, L. *Sobtop*, 1.0 (dev3.1).

10. Darden, T.; York, D.; Pedersen, L. Particle mesh Ewald: An *N*·log(*N*) method for Ewald sums in large systems. *J. Chem. Phys.* **1993,** *98*, 10089-10092.

11. Bussi, G.; Donadio, D.; Parrinello, M. Canonical sampling through velocity rescaling. *J. Chem. Phys.* **2007,** *126*, 014101.

12. Parrinello, M.; Rahman, A. Polymorphic transitions in single crystals: A new molecular dynamics method. *J. Appl. Phys.* **1981,** *52*, 7182-7190.
